# Boosting oxygen reduction activity and enhancing stability through structural transformation of layered lithium manganese oxide

**DOI:** 10.1038/s41467-021-23430-3

**Published:** 2021-05-25

**Authors:** Xuepeng Zhong, M’hamed Oubla, Xiao Wang, Yangyang Huang, Huiyan Zeng, Shaofei Wang, Kun Liu, Jian Zhou, Lunhua He, Haihong Zhong, Nicolas Alonso-Vante, Chin-Wei Wang, Wen-Bin Wu, Hong-Ji Lin, Chien-Te Chen, Zhiwei Hu, Yunhui Huang, Jiwei Ma

**Affiliations:** 1 Shanghai Key Laboratory for R&D and Application of Metallic Functional Materials, Institute of New Energy for Vehicles, School of Materials Science and Engineering, Tongji University, Shanghai, China; 2 Max Planck Institute for Chemical Physics of Solids, Dresden, Germany; 3 Australian Centre for Neutron Scattering, Australian Nuclear Science and Technology Organization, Kirrawee DC, NSW Australia; 4 Center for Alloy Innovation and Design, State Key Laboratory for Mechanical Behavior of Materials, Xi’an Jiaotong University, Xi’an, China; 5 Beijing National Laboratory for Condensed Matter Physics, Institute of Physics, Chinese Academy of Sciences, Beijing, China; 6 Songshan Lake Materials Laboratory, Dongguan, China; 7 Spallation Neutron Source Science Center, Dongguan, China; 8 IC2MP, UMR-CNRS 7285, University of Poitiers, Poitiers, France; 9 National Synchrotron Radiation Research Center, Hsinchu, Taiwan

**Keywords:** Electrocatalysis, Materials chemistry, Fuel cells

## Abstract

Structural degradation in manganese oxides leads to unstable electrocatalytic activity during long-term cycles. Herein, we overcome this obstacle by using proton exchange on well-defined layered Li_2_MnO_3_ with an O3-type structure to construct protonated Li_2-x_H_x_MnO_3-n_ with a P3-type structure. The protonated catalyst exhibits high oxygen reduction reaction activity and excellent stability compared to previously reported cost-effective Mn-based oxides. Configuration interaction and density functional theory calculations indicate that Li_2-x_H_x_MnO_3-n_ has fewer unstable O *2p* holes with a Mn^3.7+^ valence state and a reduced interlayer distance, originating from the replacement of Li by H. The former is responsible for the structural stability, while the latter is responsible for the high transport property favorable for boosting activity. The optimization of both charge states to reduce unstable O *2p* holes and crystalline structure to reduce the reaction pathway is an effective strategy for the rational design of electrocatalysts, with a likely extension to a broad variety of layered alkali-containing metal oxides.

## Abstract (web summary)

Structural degradation in manganese oxides leads to unstable activity during long-term cycles. Herein, authors demonstrated that reduced unstable O *2p* holes and the short interlayer distance of layered lithium manganese oxide are favorable for excellent electrocatalytic stability and activity.

## Introduction

The oxygen reduction reaction (ORR) plays an important role in energy conversion, spanning from polymer membrane fuel cells to metal–air batteries^1^. Sluggish ORR kinetics can be efficiently activated using platinum-based electrocatalysts. The cost and scarcity of platinum, however, have limited its practical applications. As a result, non-noble metal group electrocatalysts have received increasing attention for their practical application in energy conversion systems^2–4^. Manganese-based oxides have captured much attention because of their natural abundance, low cost, and nontoxicity^5^. Among them, MnO_2_ has been reported as a promising catalyst for the ORR in alkaline medium^6–12^, including various polymorphs. Its basic structural unit is a [MnO_6_] octahedron. The [MnO_6_] unit with corner- and edge-sharing forms MnO_2_, whose structure can be divided into three categories: one-dimensional (1D) tunneled structure (α-, β-, and γ-MnO_2_), two-dimensional (2D) layered structure (δ-MnO_2_), and three-dimensional (3D) structure (λ-MnO_2_). Based on previous reports^9, 13^, electrochemical performance of MnO_2_ depends strongly on the crystal structure and chemical composition. Among the different crystal structures of MnO_2_, δ-MnO_2_ is promising because its unique layered structure can provide a highly efficient transport pathway for the ORR^14–16^ and has a relatively high Brunauer–Emmett–Teller (BET) surface area. Nevertheless, layered manganese oxide forms a metastable structure during the electrocatalytic reaction due to the loss of active manganese ions arising from the disproportionation reaction. Its practical application is limited by these instabilities. The results of previous studies^17–20^ show that tuning the O *2p* hole concentration, induced by the structural regulation of layered manganese oxide, can be considered an effective strategy to overcome this challenge. In the case of Co^4+^ oxide (e.g., SrCoO_3_), earlier studies specify that only 8% of the 3*d*^3^ equivalent to Co^3.4+^, usually described as SrCoO_3-d_, shows stable oxygen evolution activity^18–20^. In addition, shortening the reaction path within metal oxides helps to unlock the transport property, thus boosting the catalytic activity promoting hydrogen evolution^21^.

Motivated by the abovementioned guidelines concerning the production of fewer unstable O *2p* holes to stabilize the structure, the short interlayer distance can be considered responsible for adjusting the ORR activity within layered manganese oxides. As a proof of concept, we investigated a well-defined layered model system Li_2_MnO_3_ (written in layer notation as Li[Li_0.33_Mn_0.67_]O_2_) because of its instability against oxygen release^22, 23^ and its strong hydrogen bonding^24^ through proton exchange (H_x_Li_1-x_[H_y_Li_0.33-y_Mn_0.67_]O_2-n_, denoted as HLM), which allowed us to effectively tune the O *2p* hole concentration and the interlayer distance, making the perfect venue to validate our concept. In this work, we demonstrated that the structural transition induced by proton exchange leads to decreases in unstable O *2p* holes and the interlayer distance, thereby enhancing structural stability and boosting oxygen reduction activity. This HLM catalyst exhibits excellent stability over 10,000 cycles, which is much superior to that of other reported manganese-based oxides and even comparable to that of the Mn single-atom catalyst^25, 26^. Moreover, HLM shows a high ORR activity with a larger limiting current density of −5.46 mA cm^−2^ at 1600 rpm and a higher specific activity than other reported manganese-based oxides. In addition, the half-wave potential is only ca. 60 mV less than the benchmark catalyst Pt/C. The practical application of HLM is demonstrated in a membrane-less micro laminar flow fuel cell configuration. This work highlights the beneficial use of proton exchange, which enables unlocking the activity and stability of layered alkali-containing metal oxides for the ORR, offering a paradigm for the rational design of electrocatalytic oxides for energy conversion technology.

## Results

### Structure transformation induced by proton exchange

**Fig. 1:**
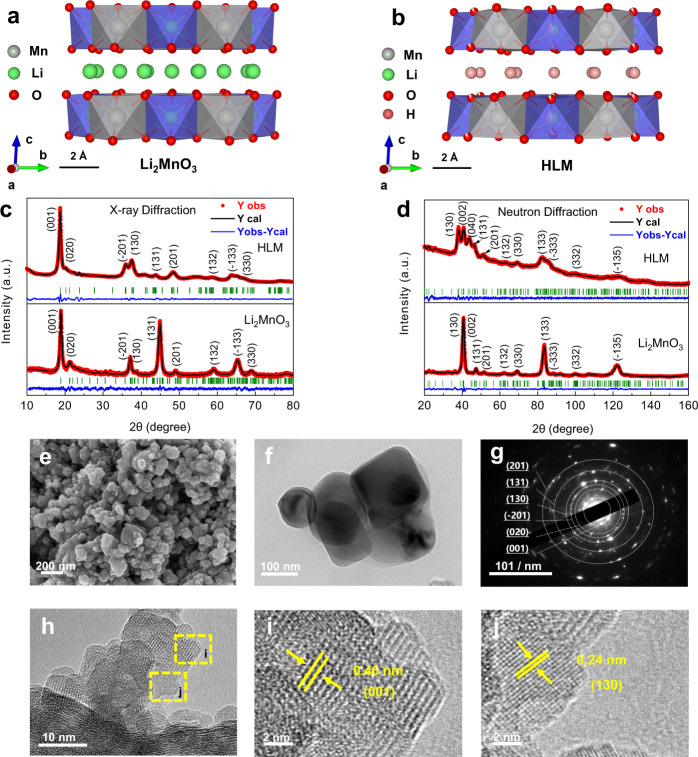
Structural characterization of Li_2_MnO_3_ and HLM. **a** Crystal structure configurations of Li_2_MnO_3_ (O3 stacking) and **b** HLM (P3 stacking). Rietveld refinement profiles using **c** X-ray diffraction and **d** neutron diffraction data for pristine Li_2_MnO_3_ (Crystallography Open Database ID: 1008373) and as-prepared HLM (Crystallography Open Database ID: 4001282) samples. The red circles illustrate the observed pattern, the black solid line is the calculated pattern, the green vertical markers show the calculated Bragg reflection positions, and the blue line is the difference between the observed pattern and the calculated pattern. **e** SEM image of HLM. **f** TEM images of HLM. **g** Electron diffraction patterns from [101] of HLM. **h** HR-TEM images of HLM. **i**, **j** HR-TEM images of selected regions of HLM.

The well-defined layered O3-type Li_2_MnO_3_ served as the soft template to build a P3-type HLM. Acid leaching was deployed to exchange lithium from thermodynamically stable Li_2_MnO_3_ and form HLM. The delithiation process was accompanied by a sharing of close-packed oxygen planes (Figs. 1a, b), resulting in a change from an O3 stacking sequence (ABC) to a P3 stacking sequence (AABBCC). The crystal structure was characterized by X-ray diffraction (XRD) analysis, which revealed the phase change upon acid leaching, identified by new peaks located at ~38° and ~49° (2θ) for HLM, as shown in Supplementary Fig. 1. The XRD pattern of HLM nearly matches the HCrO_2_ structure in the *R-3m* group. Hydrogen is located in trigonal prismatic sites within the HCrO_2_ structure, yielding strong hydrogen bonding. The broad peak located at 2700–3050 cm^−1^ in the Fourier transform infrared (FTIR) spectra indicates the presence of strong H-bonded -OH groups in the P3 structure (Supplementary Fig. 2). Strong hydrogen bonding can be further considered a driving force for the layer stacking change from O3- to P3-type structures, making the structure more stable^22^. The (001) peak of HLM shifts towards a higher 2θ compared to Li_2_MnO_3_, revealing that the interlayer spacing decreases upon the replacement of Li by H, as discussed below. The conservation of the peak located at ~21° (2*θ*) after acid leaching suggests cation ordering in the TM layers, leading to a decrease in symmetry from *R-3m* to *C2/m*^22, 24^. However, the smoothness of this peak after the delithiation process suggests cation deficiency within the TM layer^27^.

Due to the weak X-ray scattering ability of Li and H, neutron diffraction (ND) was employed to investigate the long-range structure of the Li_2_MnO_3_ and as-prepared HLM samples. As expected, joint Rietveld refinements of Li_2_MnO_3_ and HLM using XRD and ND data, shown in Fig. 1c, d and Table 1, confirmed the monoclinic structure with *C2/m* symmetry. The refined lattice parameters were *a* = 4.918(5) Å, *b* = 8.487(9) Å, *c* = 4.945(7) Å, and *V* = 195.3(9) Å^3^ for Li_2_MnO_3_ and *a* = 5.016(1) Å, *b* = 8.622(5) Å, *c* = 4.858(5) Å, and *V* = 200.2(9) Å^3^ for HLM. Similar to previous reports^24, 28^, a decrease in the *c* parameter was observed after acid leaching, while an increase in both *a* and *b* could be assigned to proton exchange in HLM. The bond distance was calculated from the refined atom position, and the shortest O–O distances between TM layers were decreased from ~3.0 Å (Li_2_MnO_3_) to ~2.5 Å (HLM), as shown in Supplementary Fig. 3. This was consistent with the strong hydrogen bonding distance^24^. Furthermore, the refined data suggest the complete extraction of Li within the Li interlayer (Li(2c) and Li(4 h)) and partial removal from the TM layer (Li(2b)). The chemical formula of HLM determined from the refined occupancies was H_1.0_[H_0.13_Li_0.17_□_0.03_Mn_0.67_]O_1.89_□_0.11_ (□ represents vacancies; Supplementary Table 1), yielding a Li/Mn ratio of ~0.254 and an average oxidation state of Mn^3.7+^ arising from oxygen release from the lattice^29–31^. The Li/Mn ratio was also determined by inductively coupled plasma-optical emission spectrometry (ICP-OES) to be ~0.25, which was consistent with the refined chemical composition. The thermogravimetric analysis (TGA) profile of HLM is shown in Supplementary Fig. 4. The calculated weight loss was ~15% due to the departure of -OH groups between 100 and 350 °C, which was close to the experimental loss of ~12%, confirming that the content of H within HLM was close to the refined chemical formula.

**Table 1:** Crystallographic details of Li_2_MnO_3_ and HLM samples obtained from joint Rietveld analysis using XRD and ND data.

Atom	Wyckoff site	x/a	y/b	z/c	B_iso_	Occup.
Li_2_MnO_3_ (space group: *C2/m*), *Z* = 4 *a* = 4.918(5) Å, *b* = 8.487(9) Å, *c* = 4.945(7) Å, *β* = 108.8(5)°, *V* = 195.3(9) Å^3 ^*χ*^2^ = 1.61, *R*_F_ = 1.36%, *R*_B_ = 2.59%, *R*_p_ = 18.7%, *R*_wp_ = 17.7%						
Mn	4g	0	0.1688 (9)	0	1.681 (4)	1.000 (5)
Li	2b	0	0.5	0	2.471 (6)	1.000 (5)
Li	2c	0	0	0.5	1.318 (6)	0.999 (8)
Li	4h	0	0.6675 (2)	0.5	0.588 (4)	0.992 (7)
O	4i	0.2286 (5)	0	0.2393 (1)	0.144 (9)	1.000 (8)
O	8j	0.2308 (5)	0.3437 (7)	0.2384 (4)	0.691 (9)	0.999 (7)
HLM (space group: *C2/m*), *Z* = 6 *a* = 5.016(1) Å, *b* = 8.622(5) Å, *c* = 4.858(5) Å, *β* = 107.7(4)°, *V* = 200.2(9) Å^3 ^*χ*^2^ = 1.5, *R*_F_ = 2.1%, *R*_B_ = 4.3%, *R*_p_ = 19.1%, *R*_wp_ = 17.4%						
Mn	4g	0	0.1746 (5)	0	2.809 (4)	1.000 (6)
Li	2b	0	0.5	0	1.280 (6)	0.516 (8)
H	2b	0	0.5	0	7.340 (8)	0.379 (5)
H	2c	0	0	0.5	1.089 (6)	0.960 (5)
H	4h	0	0.7581 (7)	0.5	1.5 (6)	0.990 (5)
O	4i	0.1171 (8)	0	−0.2111 (8)	1.679 (8)	0.876 (5)
O	8j	0.1389 (6)	0.3370 (8)	−0.2337 (6)	2.115 (9)	0.980 (3)

Figure 1e, f shows nanometric particles of the as-prepared HLM, unlike the large platelets of the precursor Li_2_MnO_3_ (Supplementary Fig. 5). The decrease in particle size was due to the “chemical grinding effect” induced by acid leaching. This was consistent with the increase in the BET surface area from ~34 m^2^ g^−1^ (Li_2_MnO_3_) to ~117 m^2^ g^−1^ (HLM) (Supplementary Fig. 6). As a result, HLM possessed a larger surface area, which could potentially provide more active sites for electrocatalytic activity. The selected area electron diffraction (SAED) pattern of the HLM showed six different diffraction rings of (001), (020), (−201), (130), (131), and (201), as shown in Fig. 1g. Figure 1h shows a high-resolution transmission electron microscopy (HR-TEM) image for HLM, demonstrating high crystallinity with interplanar distances of 0.46 nm and 0.24 nm for the (001) and (130) planes, respectively (Fig. 1i, j). These results are in good agreement with the XRD pattern observations (Supplementary Fig. 7).

### Electronic structures

**Fig. 2:**
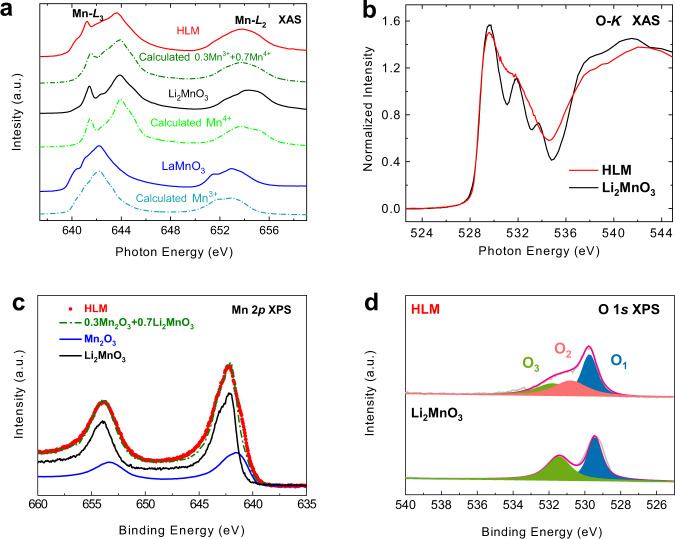
Electronic structure characterization by XAS and XPS. **a** Mn-*L*_2,3_ XAS spectra of HLM (red) together with Li_2_MnO_3_ (black) and LaMnO_3_^35^ (blue) as Mn^4+^ and Mn^3+^ references. The dashed lines below the experimental data are the corresponding calculated spectra. **b** O-*K* XAS spectra of HLM (red) and Li_2_MnO_3_ (black). **c** High-resolution Mn 2*p* XPS spectra of HLM (red) and sum (olive) of Mn_2_O_3_ (blue) and Li_2_MnO_3_ (black). **d** High-resolution O 1*s* XPS spectra of the as-prepared HLM and pristine Li_2_MnO_3_ samples.

The electronic structure was characterized by joint soft X-ray absorption spectroscopy (XAS) and X-ray photoelectron spectroscopy (XPS). The energy position and multiplet spectral feature of the XAS spectrum at the 3*d* transition metal elements *L*_2,3_ edges are highly sensitive to their valence state^32, 33^ and local environment^34^. Figure 2a shows the Mn-*L*_2,3_ XAS spectra of HLM (red) together with LaMnO_3_ (blue from Burnus et al.^35^) and Li_2_MnO_3_ (black) as a Mn^3+^ reference and a Mn^4+^ reference, respectively. The energy position of the HLM spectrum is located between LaMnO_3_ and Li_2_MnO_3_, much closer to the latter, confirming the mixed-valence states of the Mn ion. Using the configuration interaction cluster model (discussed below), we can closely reproduce the Mn^3+^ oxide (dashed line below LaMnO_3_) and Mn^4+^ oxide (dashed line below Li_2_MnO_3_). In turn, the HLM contains 30% Mn^3+^ and 70% Mn^4+^ (dashed line below HLM), giving rise to an average oxidation state of Mn^3.7+^ in HLM. The calculations were performed using CTM4XAS^36, 37^. This theory incorporates a correct absolute weight of each Mn valence state, but the energy position of each valence state has to be adjusted according to experiments from the reference materials^32, 35, 38, 39^. The spectral broadening of mixed Mn^3+^ and Mn^4+^ is due to a partial overlapping of Mn^3+^ and Mn^4+^. However, the sharp lower-energy peak (641.6 eV) at the Mn-*L*3 edge of Mn^4+^ oxide is a sensitive fingerprint for the Mn^4+^ content. The difference between HLM and Li_2_MnO_3_ is most likely related to the removal of oxygen from the lattice upon the replacement of Li by less metallic H ions, which creates Jahn–Teller Mn^3+^ ions^40^. Figure 2b presents the normalized O *K*-edge XAS spectra of Li_2_MnO_3_ and HLM. The pre-edge peaks below 533 eV are the result of O *2p* state mixing with the 3*d* transition element 3*d* state. The spectral weights of lower-energy peaks increase with increasing valence state^41^. The lowest pre-edge peak is related to the 3*d* state of the Mn^4+^ ion^33^. The loss of its spectral weight in the HLM sample with respect to that in Li_2_MnO_3_ again indicates a lower valence state in the former. A slight decrease in the Mn valence state reduces the unstable O *2p* holes close to the Fermi level and enhances the ORR stability^17^, as discussed below. Spectral broadening after the loss of oxygen can also be seen in the O-*K* XAS spectrum of HLM since the orbital character is smeared out. This indicates an increase in the transport property, which is responsible for enhancing the ORR activity of HLM. XPS was used to further characterize the change in the chemical state. Figure 2c shows the Mn 2*p* XPS spectra of HLM (red) and those of Li_2_MnO_3_ (black) and Mn_2_O_3_ (blue) as Mn^4+^ and Mn^3+^ references, respectively, which indicates that Mn exists in mixed-valence states in HLM. The ratio of Mn^3+^/Mn^4+^ determined by XPS is approximately 3:7, indicating an average oxidation state of Mn^3.7+^, which is in good agreement with Mn-*L*_2,3_ XAS and refined data from joint XRD and NPD analyses. The charge compensation for the loss of oxygen is represented by the decrease in the average valence state of manganese. As shown in Fig. 2d, the high-resolution O 1*s* XPS spectrum of Li_2_MnO_3_ exists in O1 and O3 forms (529.5 eV and 531.2 eV, respectively), corresponding to the O^2−^ peak and the oxygenated deposited species or CO_3_^2−^, respectively. In contrast, the O 1*s* signal of nonstoichiometric oxygen species (O2) for deficiencies centered at ~531 eV can be observed, further confirming the presence of oxygen vacancies within HLM^42, 43^.

### Enhanced electrochemical activity

**Fig. 3:**
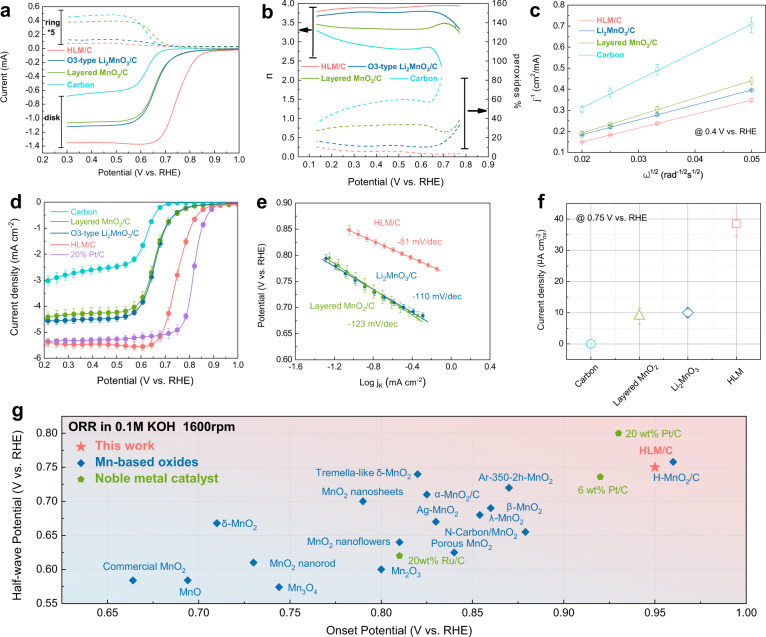
Electrochemical activity of the obtained samples in 0.1 M KOH aqueous solution. **a** ORR polarization curves of HLM/C, Li_2_MnO_3_/C, and layered MnO_2_/C and carbon at a rotation rate of 1600 rpm in O_2_-saturated 0.1 M KOH solution. **b** Electron transfer numbers (*n*) and HO_2_^−^ yields (y) for HLM/C, Li_2_MnO_3_/C, layered MnO_2_/C and carbon. **c** Koutecky-Levich plots. **d** Linear sweep voltammetry curves of HLM/C, Li_2_MnO_3_/C, layered MnO_2_/C, carbon, and 20% Pt/C obtained at O_2_-saturated 0.1 M KOH with a scan rate of 5 mV s^−1^. **e** The Tafel plots of HLM/C, Li_2_MnO_3_/C and layered MnO_2_/C. **f** Specific activities of HLM/C, Li_2_MnO_3_/C, layered MnO_2_/C and carbon. The current density at 0.75 V vs. RHE normalized by BET surface area is used as a benchmark for the comparison. **g** Comparison of HLM with other reported Mn-based oxides and noble metal catalysts. Note: (i) The onset potential is the potential at which the current density exceeds 0.1 mA cm^-2^, and the half-wave potential is the potential at which the current density is equal to half of the diffusion current density. (ii) The onset potential and half-wave potential were taken from the text or directly read from the graphics. (iii) The mass loading of reported Mn-based oxides ranges from 0.073 mg cm^-2^ to 0.465 mg cm^-2^. (iv) Error bars represent the standard deviation of at least three independent measurements.

The electrochemical activity towards the ORR was evaluated in 0.1 M KOH solution. As shown in Fig. 3a, HLM/C exhibited a half-wave potential (*E*_1/2_) of 0.75 V vs. RHE, a smaller ring current and a larger disk current in all samples (the corresponding XRD pattern of layered MnO_2_ is shown in Supplementary Fig. 8). The electron transfer number (*n*) and the HO_2_^−^ yield (y) were obtained from rotating ring-disk electrode (RRDE) measurements, as shown in Fig. 3b. Within the potential range of 0.1−0.8 V vs. RHE, for HLM/C, n was above 3.8 with HO_2_^−^ yields below ~10%. This is a value comparable to that of the benchmark Pt/C^44^. To further quantify the ORR pathway, the calculated electron transfer numbers (*n*) of HLM/C, Li_2_MnO_3_/C, layered MnO_2_/C and carbon at 0.4 V vs. RHE using Eqs. (1) and (2) were 3.99, 3.73, 3.19, and 2.00, respectively, approaching a 4-electron mechanism for the HLM/C sample, as shown in Fig. 3c. Koutecky–Levich (K–L) plots at different potential ranges of 0.2–0.5 V vs. RHE are shown in Supplementary Fig. 9. Moreover, as shown in Fig. 3d, HLM/C yielded relatively high ORR activity, with a half-wave potential of only ca. 60 mV less than 20 wt% Pt/C.

The enhanced activity can be attributed to the reduced interlayer distance and the introduction of oxygen vacancies by ion exchange from Li to H. The reduced interlayer spacing in the *c* direction helps to reduce the electrochemical reaction pathway, while the oxygen vacancies acting as active adsorption sites can improve the interaction between the HLM surface and oxygen-containing species^45^. The Tafel plots were extracted from the mass transfer corrected LSV curves, as shown in Fig. 3e. The Tafel slope of HLM/C (−81 mV/dec^−1^) differs from that of Li_2_MnO_3_/C (−110 mV/dec^−1^) and layered MnO_2_/C (−123 mV dec^−1^), suggesting that the ORR could be assigned to specific rate-determining steps that depend on the coverage and surface chemistry. If the value of the Tafel slope is ca. −60 mV dec^−1^, then the rate-determining step of the ORR is dominated by the ORR process after a one-electron transfer step. If the value of the Tafel slope is ca. −120 mV dec^−1^, the single-electron transfer step is the rate-determining step^46, 47^. Figure 3f shows the specific activities of HLM/C, Li_2_MnO_3_/C, layered MnO_2_/C, and carbon. The kinetic current density at 0.75 V vs. RHE normalized by BET surface area is used as a benchmark for the comparison^48^. HLM shows the highest current density, which is nearly four times higher than the current density of Li_2_MnO_3_. The charge transfer rate was measured by electrochemical impedance spectroscopy (EIS) in O_2_-saturated (sat.) 0.1 M aqueous KOH solution. Among these catalysts, HLM/C exhibited the smallest charge transfer resistance (*R*_ct_; Supplementary Fig. 10). *R*_ct_ followed the sequence of HLM/C < layered MnO_2_/C < Li_2_MnO_3_/C, confirming the enhanced transport property of the HLM/C catalyst. Notably, as shown in Fig. 3g, we compared HLM/C with other state-of-the-art Mn-based oxides and noble metal catalysts in terms of half-wave potentials and onset potentials (Supplementary Table 2). HLM/C shows a high ORR activity, which outperforms most of the reported Mn-based oxides.

### High stability

**Fig. 4:**
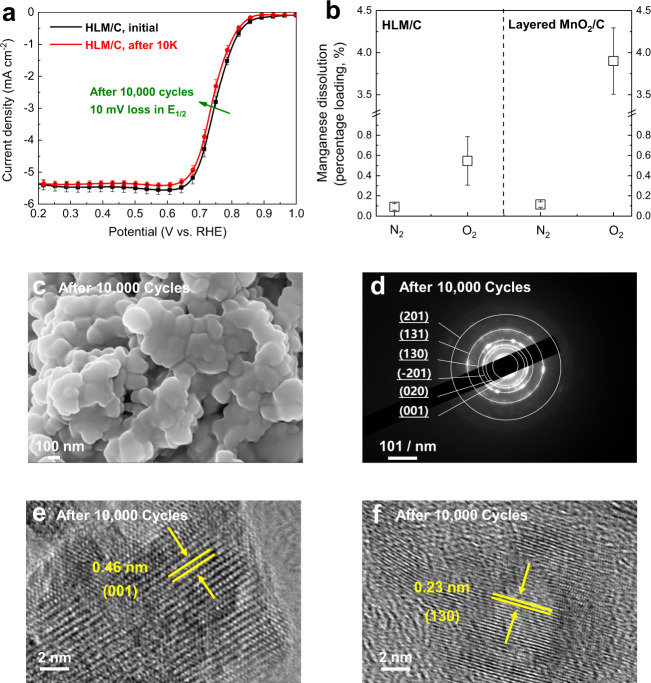
Stability characterization and ion dissolution behaviors. **a** Stability studied by using potential cycling of HLM/C. **b** Total amount of Mn dissolution studied by ICP for HLM/C and layered MnO_2_/C. Error bars represent the standard deviation of at least three independent measurements. **c** SEM image of HLM after 10,000 cycles. **d** SAED of HLM after 10,000 cycles. **e**, **f** Corresponding HR-TEM images with selected regions of HLM after 10,000 cycles.

The electrochemical stability of HLM/C was examined by accelerated cyclic voltammetry over 10,000 cycles from 0.6 V to 1.0 V vs. RHE in O_2_-saturated 0.1 M KOH. The catalyst was further subjected to the LSV test after 10,000 cycles, which showed excellent long-term stability (Fig. 4a), as evidenced by a 10 mV loss of *E*_1/2_ over 10,000 cycles, with a negligible decrease in the limiting current density over 10,000 cycles. The HLM/C catalyst outperformed the Li_2_MnO_3_/C, layered MnO_2_/C and Pt/C (20 wt% Pt/C, Johnson-Matthey) catalysts (Supplementary Fig. 11). A higher E_1/2_ loss was observed for Li_2_MnO_3_/C during repeated CV cycles. This loss was 24 mV after 10,000 cycles, and was concomitant with a slight decrease in the limiting current density. From the point of view of the electronic structure, the P3-type HLM catalyst has fewer unstable O *2p* holes near the Fermi level with a Mn^3.7+^ valence state than Li_2_MnO_3_, arising from the charge redistribution between the O *2p* and Mn *3d* orbitals. Importantly, the decrease in unstable O *2p* holes is responsible for the structural stability. On the other hand, the *E*_1/2_ and limiting current density of layered MnO_2_/C decreased rapidly during repeated CV cycles, indicating the electrochemical instability of its layered structure, with its 28 mV loss of *E*_1/2_ and 13% loss in the limiting current density after 10,000 cycles. Indeed, it was proposed that the reduction of O_2_ initiates with the reduction of Mn^4+^ to Mn^3+^ (Mn^4+^(s) + e^−^ → Mn^3+^(s)), followed by the oxidation of Mn^3+^ (Mn^3+^(s) + O_2_(ads) → Mn^4+^(s) + O_2_^−^(ads))^49–52^. Unfortunately, Mn^3+^ in the octahedral environment is unstable due to its high-energy unpaired single electron, resulting in self-stabilization that might be caused by three reaction mechanisms, namely, Jahn-Teller distortion, ORR, and disproportionation reaction^49^. Previous studies have confirmed that the incorporation of Li within the TM layer allows the strengthening of the TM-O bonds as a result of compensation for the loss of the O effective coordination number^53^. The increased TM-O bond strength compared to Mn–O leads to the suppression of the disproportionation reaction of Mn^3+^ to Mn^4+^ and Mn^2+^. To confirm this, ICP-OES was utilized to further characterize metal dissolution in both N_2_-sat. and O_2_-sat. electrolytes after the stability test, as shown in Fig. 4b. In N_2_-sat. electrolyte, HLM/C and layered MnO_2_/C exhibit a similar negligible dissolution of Mn (~0.09% and ~0.11% of Mn dissolved for HLM/C and layered MnO_2_/C, respectively). However, the presence of O_2_ in the electrolyte causes increased dissolution of manganese. For layered MnO_2_/C, ~3.9% of Mn was dissolved after 10,000 cycles in the O_2_-sat. electrolyte. In contrast, the dissolution of the corresponding Mn in HLM/C was only ~0.55%. It is generally accepted that inducing Li within the TM layer plays an important role in stabilizing the structure^54^. The mixing of low valent Li^+^ and high valent Mn^4+^ can effectively immobilize adjacent oxygen layers, thereby further improving electrochemical stability. Maintaining the lithium ions in octahedral sites within the TM layer is the key factor for stabilizing the layered structure over the electrocatalytic reaction process.

The morphology of HLM remained basically unchanged, as shown in Fig. 4c. The SAED pattern showed that the structure of HLM was sustained over 10,000 potential cycles (Fig. 4d). This result was further confirmed by the interplanar spacing of 0.46 nm and 0.23 nm for the (001) and (130) planes of HLM after 10,000 cycles of testing (Fig. 4e, f), which were similar to those of the as-prepared HLM before stability measurement. All these results demonstrated that HLM/C is stable over a long-term electrocatalytic reaction process. Compared to other reported electrocatalysts, HLM/C shows a high stability (Supplementary Table 3).

## Discussion

### Theoretical description of electronic structures and mechanistic insights

To better understand the origin of the excellent ORR activity and stability, configuration interaction (CI) clustering and density functional theory (DFT) calculations were performed. In the CI calculations, the ground-state wave function of the late 3*d* transition metal oxides (TM) is usually described as Φ_g_ = α| 3*d*^n^ 〉 + β|3*d*^n+1^*Ḻ* 〉 + γ|3*d*^n+2^*Ḻ*^2^〉^55, 56^, where *Ḻ* denotes an oxygen 2*p* hole, and *α*, *β*, and *γ* are linear combination coefficients to construct an orthonormal wave function Φ_g_. The content of O *2p* holes, namely, the |3*d*^n+1^*Ḻ*〉 and |3*d*^n+2^*Ḻ*^2^〉 configurations, increases with decreasing charge transfer energy ∆. In general, ∆ decreases with increasing valence of TM. In turn, the 3*d*^n^ configuration decreases from ~90–95% for divalent 3*d* elements with ∆ ~ 7 eV^55, 57^ to only 10% for Co^4+^ with ∆ ~ −4 eV^41, 56^. Note that ∆ decreases slightly with increasing atomic number for the later 3*d* transition metals from Mn to Ni through Fe and Co.

Therefore, the O *2p* holes in the charge transfer insulators mainly originate from TM *3d* and O *2p* covalence (O–O bonding), and Δ is a crucial value for the redox activity and structure stability. Using the configuration interaction cluster model, the experimental Mn-*L*_2,3_ XAS spectrum of Li_2_MnO_3_ (black line) and LaMnO_3_ (blue line) can be nicely reproduced (dashed lines below experimental data in Fig. 2a). The parameters values are 10Dq = 2.0 eV and Δ = − 1.0 eV for Mn^4+^ and 10Dq = 1.0 eV, Ds = 0.21 eV, Dt = 0.03 eV, and Δ = 4.0 eV for Mn^3+^^17^.

The Mn *3d* to O *2p* transfer integrals were calculated via Harrison’s prescription on the basis of the Mn–O bond lengths, and the interatomic multiplet interaction (the Slater integrals) was reduced to 75% of the atomic values. The theory yields only approximately 20% 3*d*^3^ for Mn^4+^ ions^17^, which means that the dominant configuration for the Mn^4+^ state in the ground state is 3*d*^4^*L*. However, the degree of covalence is smaller than that of Co^4+^, which has only 8% 3*d*^5^ configuration in the ground state^41, 56^. For the Mn^3+^ ion, our CI theory gives ~60% 3*d*^4^ and a small amount of 3*d*^5^*L* configuration, namely, much fewer O *2p* holes. As a next step, the experimental Mn-*L*_2,3_ XAS spectrum (red line) of HLM was reproduced by 30% Mn^3+^ and 70% Mn^4+^ (dashed olive line below the experimental data in Fig. 2a).

The partial high oxidation state gives rise to high electrochemical performance^40^. Further oxidation of the lattice oxygen leads to the formation of O-O bonds, which was directly observed in the RIXS and XAS spectra at the O-*K* edge for the stable O *2p* holes^58^. However, the excessive number of O *2p* holes in the high oxidation state is disadvantageous to the structural stability since unstable O *2p* holes lead to O_2_ release under electrochemical conditions^17, 59–62^. Oxygen release is well known to occur in Co^4+^ oxide ACoO_3_ (A = Ca and Sr) due to unstable O *2p* holes. The stable material is SrCoO_2.7_, possessing Co^3.4+^^18–20^. Considering that the total O *2p* holes in Mn^4+^ oxides are only half of those in Co^4+^ oxides, the stable valence state of the Mn ion could be in the range of 3.7+ and 3.8+, which is consistent with our experimental results in the HLM sample. Here, our results are consistent with previous findings indicating that the stability of the crystal structure increases from the Mn^4+^ state in LiNi_0.5_Mn_1.5_O_4_ to Mn^3.67+^ in LiNi_0.2_Mn_1.8_O_4_^17^. The occurrence of Mn^3+^ ions in the Mn^4+^ lattice favors its transport property through the double exchange (DE) mechanism developed by Zener^63–65^. In the DE scenario, electrons in a ligand (O) between ions with different oxidation states (Mn^3+^ and Mn^4+^) could easily hop (through Mn^3+^–O and O–Mn^4+^), realizing a delocalized state with reduced kinetic energy. Hence, the transport property would be boosted, as observed in our experiments.

**Fig. 5:**
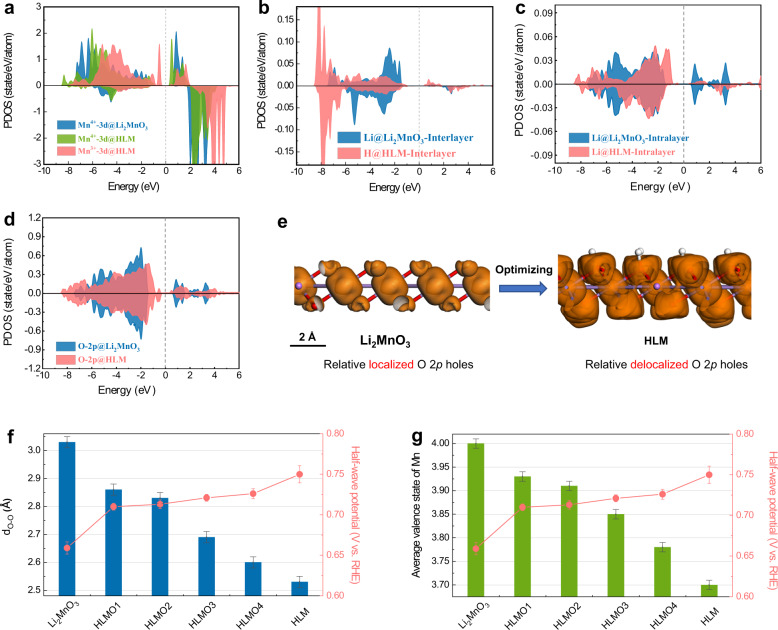
Theoretical description and mechanistic study. Comparison of the PDOS of Li_2_MnO_3_ and HLM systems with **a** Mn-*d* orbitals, **b** interlayer Li-*s* (in Li_2_MnO_3_) and H-*s* (in HLM), **c** intralayer Li-*s*, and **d** O-*2p* orbitals. The energy is shifted relative to the Fermi level of HLM. **e** Band decomposed charge density of the lowest conduction band (between 0 and 2 eV above the Fermi level) before and after proton exchange. Relationships of **f** interlayer distances and **g** Mn valence states versus ORR activity with respect to half-wave potentials. Error bars represent the standard deviation of at least three independent measurements.

Although our CI calculations could naturally present the numbers of 3*d* occupations and O *2p* holes within the MnO_6_ cluster, it was necessary to go beyond the cluster to understand transport properties. For this purpose, we turned to DFT calculations. Considering the strong correlation of the Mn-*d* orbitals, we adopted the DFT + *U* method with an effective *U* value of 4 eV^66, 67^. Note that even though the DFT + *U* scheme is widely adopted to incorporate strong correlation, it still uses the framework of a single-electron approximation and requires comparisons with experimental observations (see Supplementary Note 2 for detailed discussions). The calculated projected density of states (PDOS) values of the Li_2_MnO_3_ and HLM models are shown in Fig. 5a–d. Overall, the band gap is reduced in the HLM system compared with that in Li_2_MnO_3_. Mn-*d* and O-*p* orbitals are the main contributors to the band gap of HLM. In the HLM system with O vacancies, there are two types of Mn ions, namely, Mn^4+^ and redox Mn^3+^, and the latter is adjacent to an O vacancy. From Fig. 5a, the PDOS peak positions of Mn^4+^ in the HLM remain similar to those in Li_2_MnO_3_. When the interlayer Li ions are fully replaced by H, we find that these H atoms tend to bind to O, leaving a channel of ~2.5 Å between neighboring layers, which is consistent with the refined structural data. This effect could further facilitate ion transport. In the redox HLM system, the simultaneous effects of electron hopping between Mn^4+^ and Mn^3+^ (DE mechanism) and the reduced band gap could enhance transport, which was consistent with the experimental observations.

If the transition metals are highly oxidized, excessive O *2p* holes exist, which are on average located at ~1.1 eV in Li_2_MnO_3_. In the redox HLM system, the total number of these O *2p* holes decreases, and their energy level increases slightly with an average value of ~1.2 eV. Figure 5d shows that the lowest unoccupied O *2p* state above the Fermi level in HLM is reduced with respect to that of Li_2_MnO_3._ This agrees well with the experimental O-*K* XAS spectra (Fig. 2b). We further plot the band decomposed charge density of the lowest conduction band between 0 and 2 eV above the Fermi level before and after the replacement of Li by H (Fig. 5e). These O *2p* holes are relatively localized in Li_2_MnO_3_ and become relatively delocalized in HLM, which is consistent with the more expanded conduction band energy in the PDOS plot. By integrating these conduction band states, the total number of O *2p* holes is reduced from Li_2_MnO_3_ to HLM, leading to improved structural stability in the latter.

To further elucidate how the interlayer distances and the ratios of Mn^3+^/Mn^4+^ affect the optimization of ORR activity, we prepared control samples with different interlayer distances (Supplementary Fig. 12, Supplementary Table 4, and Supplementary Table 5) and Mn^3+^/Mn^4+^ ratios (Supplementary Fig. 13 and Supplementary Table 6) and further assessed their ORR performance (Supplementary Fig. 14). Indeed, the degree of protonation can be adjusted with different acid concentrations, and the ratios of Li/Mn after acid leaching can be determined by ICP-OES. Notably, the phase transition from O3 to P3 stacking occurred as the acid concentration increased to 0.0642 M H_2_SO_4_ (HLMO3, Supplementary Fig. 12). The extracted Li number was ~1.58 (higher than the 1.5 interlayer Li number in Li_2_MnO_3_). This can trigger the phase transition. Figure 5f shows that the reduced interlayer distances yield different degrees of enhancement of ORR activity with respect to E_1/2_: Li_2_MnO_3_ (0.659 V vs. RHE) < HLMO1 (0.710 V vs. RHE) < HLMO2 (0.713 V vs. RHE) < HLMO3 (0.721 V vs. RHE) < HLMO4 (0.726 V vs. RHE) < HLM (0.75 V vs. RHE). Similar trends were observed, and the ORR activity was enhanced with increasing Mn^3+^ ratio in the Mn^4+^ lattice, confirming that a favorable transport property through the DE mechanism is responsible for boosting the ORR activity, as shown in Fig. 5g.

### Broader impact of generality and micro laminar flow fuel cell application

**Fig. 6:**
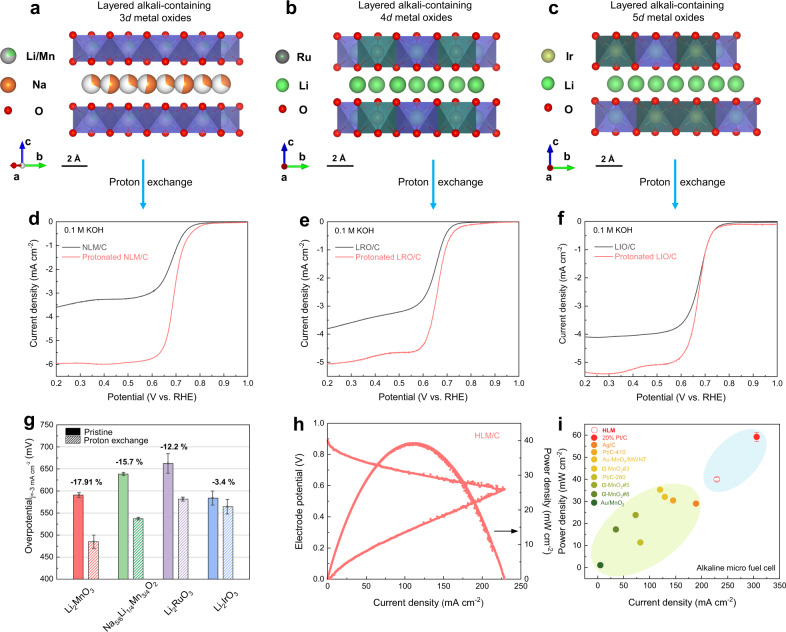
Generality of concept and micro laminar flow fuel cell performance. Crystal structure configurations of **a** layered Na-containing 3*d* metal oxide, **b** layered Li-containing 4*d* metal oxide and **c** layered Li-containing 5*d* metal oxide. **d**–**f** ORR activity of layered alkali-containing (Li and Na) metal (from 3*d* to 5*d*) oxides measured in 0.1 M KOH. **g** The overpotentials of Li_2_MnO_3_, Na_5/6_Li_1/4_Mn_3/4_O_2_, Li_2_RuO_3_, and Li_2_IrO_3_ at a current density of −3 mA cm^−2^ before and after proton exchange. **h** Current-potential characteristics and corresponding power density curves of HLM in H_2_/O_2_-µLFFCs. **i** Comparison of the HLM with other reported catalysts in alkaline-based micro fuel cells in terms of current density and power density. Error bars represent the standard deviation of at least three independent measurements.

To further generalize the concept to a broader class of layered alkali-containing metal oxide materials, three layered alkali-containing metal oxides, namely, Na-containing 3*d* metal oxides Na_5/6_Li_1/4_Mn_3/4_O_2_ (denoted by NLM; Fig. 6a), Li-containing 4*d* metal oxides Li_2_RuO_3_ (equivalently, Li[Li_0.33_Ru_0.67_]O_2_, denoted by LRO; Fig. 6b), and Li-containing 5*d* metal oxides Li_2_IrO_3_ (equivalently, Li[Li_0.33_Ir_0.67_]O_2_, denoted by LIO; Fig. 6c), were investigated. Notably, an enhancement of the electrochemical performance was observed for layered alkali-containing (Li and Na) metal (from 3*d* to 5*d*) oxides upon the replacement of lithium and/or sodium with small H, demonstrating the generality of our concept (Fig. 6d–f). The overpotential was extracted at a current density of −3 mA cm^−2^ as a benchmark for the sake of comparison, as shown in Fig. 6g. Proton exchange within Li_2_MnO_3_ leads to an increase in electrocatalytic activity, yielding a 17.9% decrease in overpotential, whereas there is a 15.7% decrease for Na_5/6_Li_1/4_Mn_3/4_O_2_. Furthermore, a similar decreasing trend was observed for Li-rich 4*d* and 5*d* metal oxides, i.e., Li_2_RuO_3_ and Li_2_IrO_3_, yielding a 12.2% decrease in overpotential for Li_2_RuO_3_ and 3.4% for Li_2_IrO_3_, respectively. Proton exchange improved the ORR activity of alkali-containing metal oxide materials, providing a strategy that enables unlocking the catalytic activity.

The practical application of the as-prepared HLM catalyst in a micro laminar flow fuel cell (µLFFC) was demonstrated. The as-prepared HLM was employed as a cathode electrocatalyst in H_2_/O_2_-µLFFC. The current-potential j-E characteristics and corresponding power density curves of HLM in the H_2_/O_2_-µLFFC are shown in Fig. 6h. The open-circuit voltage of the P3-type HLM catalyst was ~0.91 V, rather close to that of Pt/C (20 wt% Pt, Johnson-Matthey, 0.95 V)^68^. In addition, excellent cell performance was obtained for the HLM with ~40 mW cm^−2^, reaching ~70% of the power density using Pt/C in the same system (Supplementary Fig. 15). As shown in Fig. 6i, a comparison of the performance for HLM, 20 wt% Pt/C, and other reported catalysts in alkaline-based micro fuel cell systems (see details in Supplementary Table 7) confirmed the superior performance of the HLM/C catalyst.

In summary, we improved the ORR activity and stability of layered manganese-based oxides through the construction of a P3-type HLM electrocatalyst by facile acid leaching on a well-defined layered O3-type Li_2_MnO_3_ material. The improved performance of the HLM electrocatalyst was evidenced by an improvement in the kinetic current density nearly four times higher than that of Li_2_MnO_3_, with almost no structural degradation over 10,000 potential cycles. The enhanced ORR performance of the HLM catalyst was related to (i) the fewer unstable O *2p* holes arising from the decreased manganese valence and the presence of Li/Mn mixing in the TM layer, which can stabilize the layer structure; (ii) the interlayer distance reduction and the occurrence of Mn^3+^ in the Mn^4+^ lattice concomitant with oxygen vacancies, which can unlock a short interlayer pathway and high transport. We have shown that the optimization of both the charge state and crystalline structure boosts the catalytic activity and the stability of layered manganese-based oxides towards the ORR. The use of the proton exchange method for the rational design of advanced electrocatalysts can exploit a broad range of alkali-containing metal (from 3*d* to 5*d*) oxide materials towards the ORR for energy conversion technology.

## Methods

### Materials synthesis

Rock salt monoclinic O3-type Li_2_MnO_3_ was prepared by a solid-state reaction. The recovered powder was further treated by acid leaching (2.5 M H_2_SO_4_) to obtain a P3-type layered HLM. There was no specific preparation procedure for the samples. For the sake of comparison, layered birnessite δ-MnO_2_ was prepared. More details can be found in the Supplementary Information (Supplementary Note 1).

### Physical characterization

The structure of the catalysts was characterized by X-ray diffraction (XRD) with a Bruker D8 Advance powder diffractometer (operating at 40 kV, 40 mA) equipped with a Cu−K_α_ source (*λ*1 = 1.5405 Å, *λ*2 = 1.5443 Å) and fitted with a beryllium window at room temperature. The neutron diffraction (ND) data were collected on an Echidna high-resolution neutron powder diffractometer with a wavenumber of 1.615 Å at the Australian Centre for Neutron Scattering. The crystal structures were illustrated using VESTA software. Joint Rietveld refinements for XRD and ND datasets were performed with the Full-Prof program, and the refined parameters were background parameters, line shift errors (zero shift), Caglioti coefficients (U, V and W), scale factor, lattice parameters, atomic position, atomic rate occupancy and isotropic atomic displacement parameters. The procedure for the corefinements (XRD and ND) was similar to regular XRD or ND data refinement. The only difference was that the structural model was matched with both XRD and ND datasets.

The electronic structure was characterized using X-ray photoelectron spectroscopy (XPS) on a Thermo Scientific Escalab 250Xi with an Al-K_α_ source. Soft X-ray absorption spectroscopy (XAS) experiments were performed at the 11 A beamline at the National Synchrotron Radiation Research Center (NSRRC) in Taiwan. Single crystals of MnO and NiO were measured simultaneously in a separate chamber as energy references for the Mn-*L*_2,3_ and O-*K* edges, respectively.

The morphology of the catalysts was determined by scanning electron microscopy (SEM) on a Zeiss Supra 55 at an acceleration voltage of 5 kV. High-resolution transmission electron microscopy (HR-TEM) and selected area electron diffraction (SAED) data were collected on a JEOL 2100 F instrument at a working voltage of 200 kV. The N_2_ adsorption/desorption isotherms were recorded on a Micromeritics TriStar II 3020 at 77 K. The stoichiometry of Li and Mn was determined, and metal dissolution in the cycled electrolyte was analyzed using inductively coupled plasma-optical emission spectrometry on an Agilent ICP-OES 730. Briefly, a dispersed catalyst suspension was drop-casted onto a polished glassy carbon disk and dried. The 0.1 M KOH electrolyte was purged with N_2_ or O_2_. After the electrolyte was N_2_ or O_2_-saturated, the drop-coated glassy carbon was used as a working electrode for long-term durability experiments over 10,000 cycles. After the durability test, the metal dissolution in the cycled electrolyte (pH was adjusted to 2–4 before analyses) was analyzed using inductively coupled plasma-optical emission spectrometry.

### Electrochemical characterization

For electrochemical measurements, a standard three-electrode system with a rotator (Pine Research Instrumentation, USA) was used on a Bio-Logic multichannel workstation (VMP3, Bio-Logic, France). To ensure electrical conductivity, the prepared catalysts were mixed with carbon powders (Vulcan XC-72R), and the mass ratio of carbon to catalyst was 70%:30%. The obtained catalysts (HLM/C, Li_2_MnO_3_/C, layered MnO_2_/C) containing 3 mg of as-prepared HLM, Li_2_MnO_3_, layered MnO_2_ and 7 mg of carbon powder (Vulcan XC-72R) were dispersed in a solution of Nafion (0.05 ml, 5 wt% in isopropanol, Aldrich), ethanol (1.01 ml) and deionized (DI) water (0.1 ml), and the resulting ink was ultrasonicated for 10 min. The evenly dispersed catalyst ink was applied to the surface of a prepolished glassy carbon disk and dried at room temperature to form a catalyst film. The mass loading of the obtained catalytic Mn-based samples was ~0.12 mg cm^−2^. For comparison, 20 wt% Pt/C (Johnson-Matthey) was purchased and studied. The three-electrode cell system used Hg/HgO as the reference electrode, graphite as the counter electrode, and the rotating (ring-)disk electrode as the working electrode (GC electrode, 5.61 mm in diameter). A solution of 0.1 M KOH was used as alkaline medium. The reference electrode was transferred to the reversible hydrogen electrode (RHE) using E (vs. RHE) = E (vs. Hg/HgO) + 0.0591 pH + 0.098 V. The electrochemical data were iR-compensated. Before the collection of electrochemical data, cyclic voltammetry (CV) was performed to clean the catalyst at a scan rate of 100 mV s^−1^ for 50 cycles. Subsequently, CV was tested at a rate of 20 mV s^−1^ in the potential range between 1.2 V and 0.05 V vs. RHE. Linear sweep voltammetry (LSV) was conducted at a sweep rate of 5 mV s^−1^. The background current was corrected by subtracting the current in the N_2_-sat. electrolyte from that in the O_2_-sat. electrolyte. To determine the catalyst stability, potential cycling was performed between 0.6 and 1.0 V vs. RHE at 100 mV s^−1^ in both O_2_-sat. and N_2_-sat. 0.1 M KOH electrolyte.

The electron transfer number (*n*) during the ORR was calculated by the Koutecky–Levich (K–L) equations:^69^

1 $$\frac{1}{j}=\frac{1}{{j}_{{\mathrm{L}}}}+\frac{1}{{j}_{{\mathrm{K}}}}=\frac{1}{B{\omega }^{\frac{1}{2}}}+\frac{1}{{j}_{{\mathrm{K}}}}$$

2 $$B=0.2{nF}{C}_{0}{D}_{0}^{\frac{2}{3}}{\nu }^{\frac{-1}{6}}$$

where *j*, *j*_L_, and *j*_k_ correspond to the measured, diffusion-limiting, and kinetic current densities, respectively; *ω* is the rotation rate (rpm), is the Faraday constant (96485 C mol^−1^), is the bulk concentration of oxygen (1.26 × 10^−6^ mol cm^−3^), is the diffusion coefficient of oxygen (1.9 × 10^−5^ cm^2^ s^−1^); and is the kinetic viscosity (0.01 cm^2^ s^−1^).

The rotating ring-disk electrode (RRDE) approach was utilized to further determine the electron transfer number (*n*) and byproduct peroxide species (*y*). The potential for the Pt ring was set at 1.48 V vs. RHE. The calculations were made with the following formula:^70^

3 $$n=4N{I}_{{\mathrm{D}}}/(N{I}_{{\mathrm{D}}}+{I}_{{\mathrm{R}}})$$

4 $$y=200{I}_{{\mathrm{R}}}/(N{I}_{{\mathrm{D}}}+{I}_{{\mathrm{R}}})$$

where *I*_D_ and *I*_R_ are the disk current and ring current, respectively. *N* = 0.37 is the RRDE collection efficiency.

### Computational methods

We performed density functional theory calculations with generalized gradient approximation (GGA) treatment of the exchange correlation functional in the Perdew–Burke–Enzerhof (PBE) form, as implemented in the Vienna ab initio simulation package (VASP)^71–73^. The projector augmented wave method and planewave basis set were adopted to treat the core and valence electrons, respectively. To correct for the strong correlation of magnetic Mn-*d* orbitals, we adopted the DFT + *U* method with an effective *U* value of 4 eV^66, 67^. The cutoff energy for the planewave basis set was set to 500 eV. In our model, we used a simulation supercell containing 4 Mn, 12 O, and 8 Li atoms for Li_2_MnO_3_. For HLM, a larger supercell with 12 Mn, 21 H, 3 Li, and 34 O was used to incorporate ~5.5% O vacancies, consistent with the refined chemical composition. Various *U* values for O *2p* orbitals (when O vacancies were presented) were also tested, and marginal differences were obtained when *U* was not applied (Supplementary Note 2 and Supplementary Fig. 16). Thus, in the main text, no on-site energy of O *2p* orbitals were used. The first Brillouin zone was represented using Monkhorst-Pack *k*-mesh with a grid density of 2*π* × 0.02 Å^–1^. These structures were fully relaxed without any symmetry constraints until the total energy and force converged within 10^−6^ eV and 0.01 eV/Å, respectively. We set the energy relative to the Fermi level of HLM, which was determined by the Fermi-Dirac distribution of electrons and holes at room temperature.

### Micro laminar flow fuel cell characterization

The H_2_/O_2_ micro laminar flow fuel cell experiment was conducted by using HLM/C (2.4 mg_HLM_ cm^-2^) as the cathode and 20 wt% Pt/C (0.8 mg_Pt_ cm^-2^, Johnson-Matthey) as the anode in an alkaline medium. Typically, a physical mixture (70 wt%:30 wt%) of Vulcan carbon (XC-72R) and HLM was dispersed in a solution containing water, isopropanol and 5 wt% Nafion by ultrasound for 2 h. Each ink was nitrogen-sprayed (2 Bar max) on carbon paper (Toray Teflon Treated carbon paper, TGP-H-900, fuel cell store) and heat-treated at 90 °C. After spraying the suspension, the plate was removed 10 min later. The carbon paper was cooled to room temperature and then pressed between Teflon plates at 500 psi for 1 min. In the H_2_/O_2_ micro laminar flow fuel cell (H_2_/O_2_-µLFFC), a 3 M KOH electrolyte stream was introduced in the one-system channel from the bottom to the top at a flow rate of 9 cm^3^ min^−1^ using a micropump (Miniplus 3 Gilson). All measurements were performed at 25 °C with H_2_/O_2_ under the same pressure of ca. 1 Bar.

## Supplementary information

Supplementary Information

## Data Availability

All relevant data are available from the corresponding authors on request. Source data are provided with this paper.

## Source data

Source Data
