# Supplementary material for: Boosting oxygen reduction activity and enhancing stability through structural transformation of layered lithium manganese oxide
Source: Nat Commun. 2021 May 25;12:3136. doi: 10.1038/s41467-021-23430-3 (PMC8149866; doi:10.1038/s41467-021-23430-3)
Supplement: Supplementary file 1 — Supplementary Information [file 41467_2021_23430_MOESM1_ESM.pdf]

Boosting Oxygen Reduction Activity and Enhancing Stability through Structural Transformation of Layered Lithium Manganese Oxide

Zhong *et al.*

Supplementary Section 1. Physical characterization.

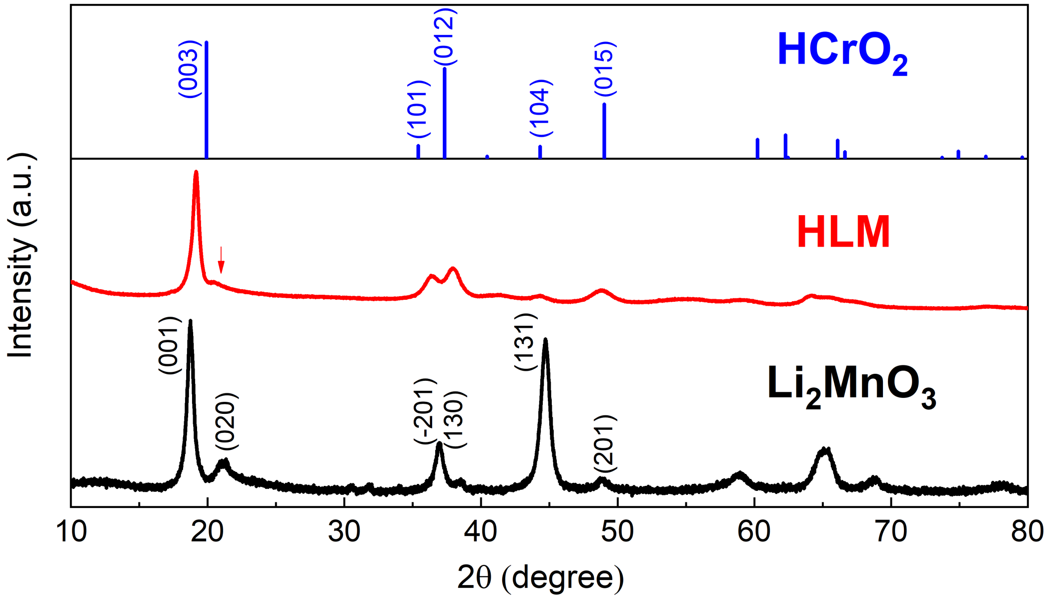

Supplementary Figure 1. XRD patterns of Li_2MnO_3 and HLM samples. A reference structure is given by $HCrO_2$.

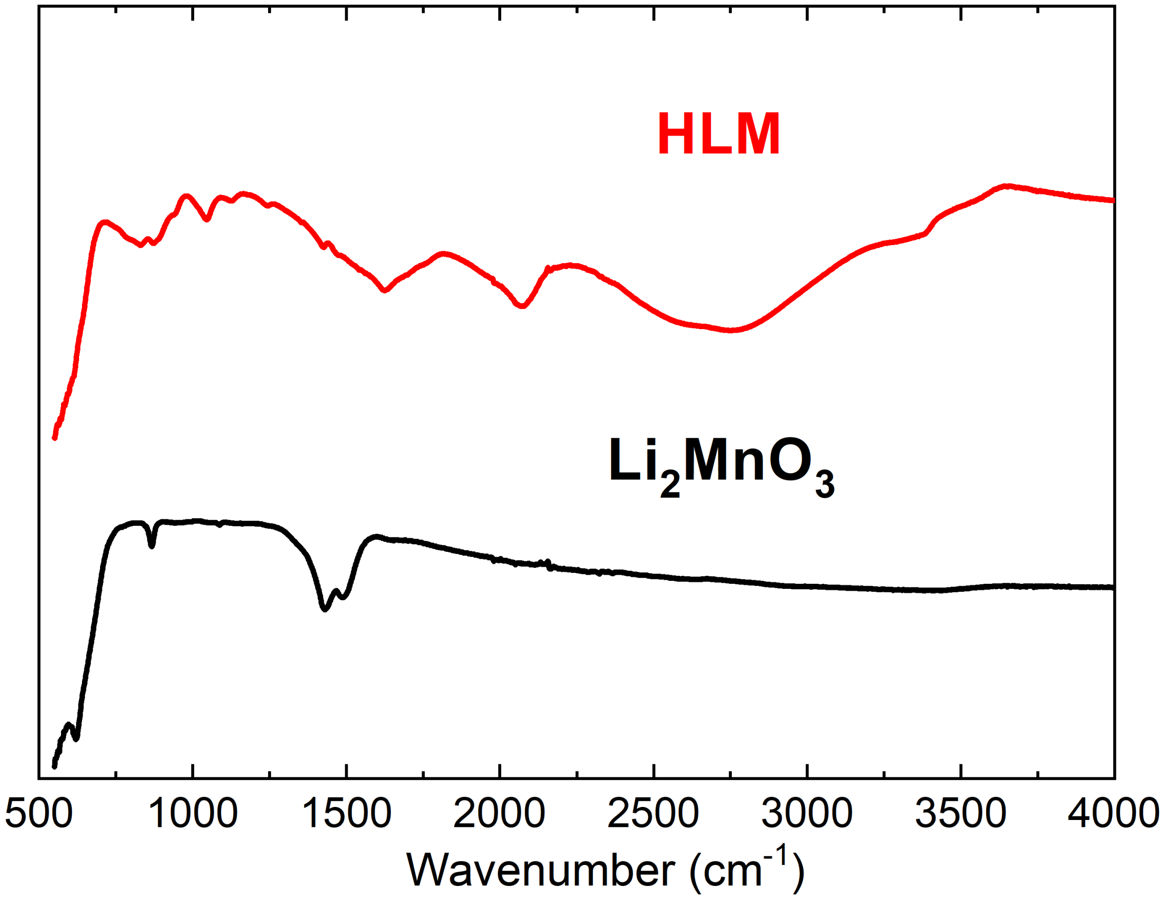

Supplementary Figure 2. Fourier transform infrared (FTIR) spectra of pristine Li_2MnO_3 and HLM.

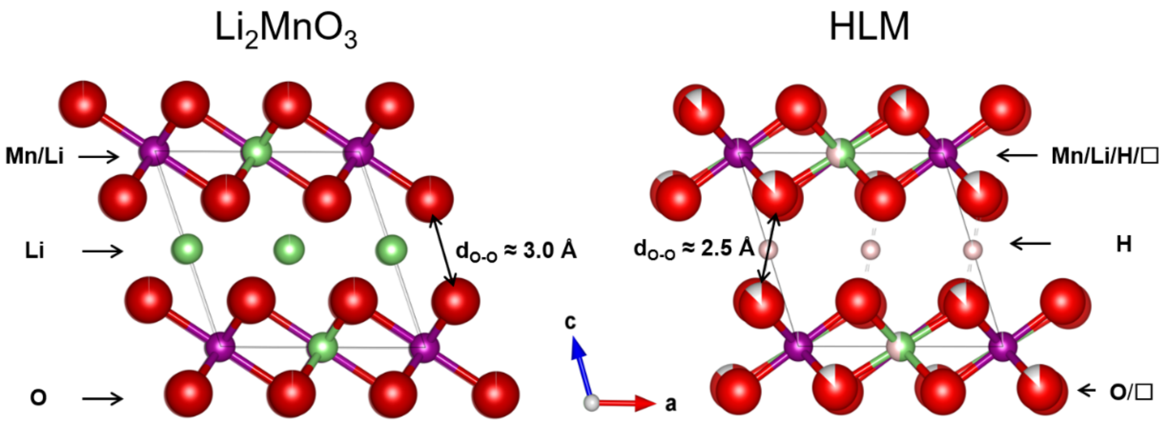

Supplementary Figure 3. Refined crystallographic structural configurations of Li_2MnO_3 and HLM samples.

Supplementary Table 1. Wyckoff sites, Position Multiplicity (*M*), Occupancy and Stoichiometric Indices (*n*) of each atom for HLM (Z=6).

Atoms	Wyckoff sites	Position Multiplicity (*M*)	Occupancy≤1	Stoichiometric Indices (*n*)*
Mn	4g	4	1.000(6)	0.67
Li	2b	2	0.516(8)	0.17
H (1)	2b	2	0.379(5)	0.13
H (2)	2c	2	0.960(5)	1.0
H (3)	4h	4	0.990(5)	
O (1)	4i	4	0.876(5)	1.89
O (2)	8j	8	0.980(3)	

*The stoichiometric indices for the HLM were calculated according to the equation $n=M\times\text{Occupancy}/Z$, where *M* is Wyckoff Position Multiplicity, Z is the number of formula units in the unit cell.

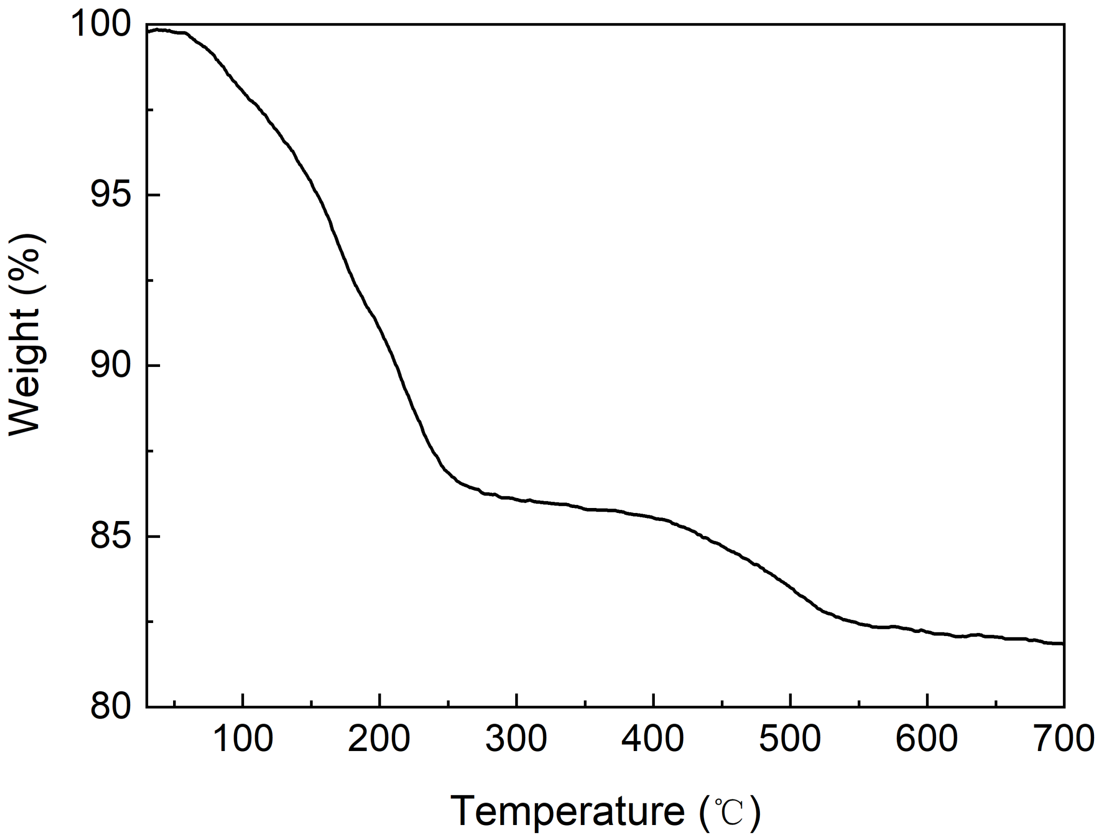

Supplementary Figure 4. The thermogravimetric (TG) analysis curve of HLM.

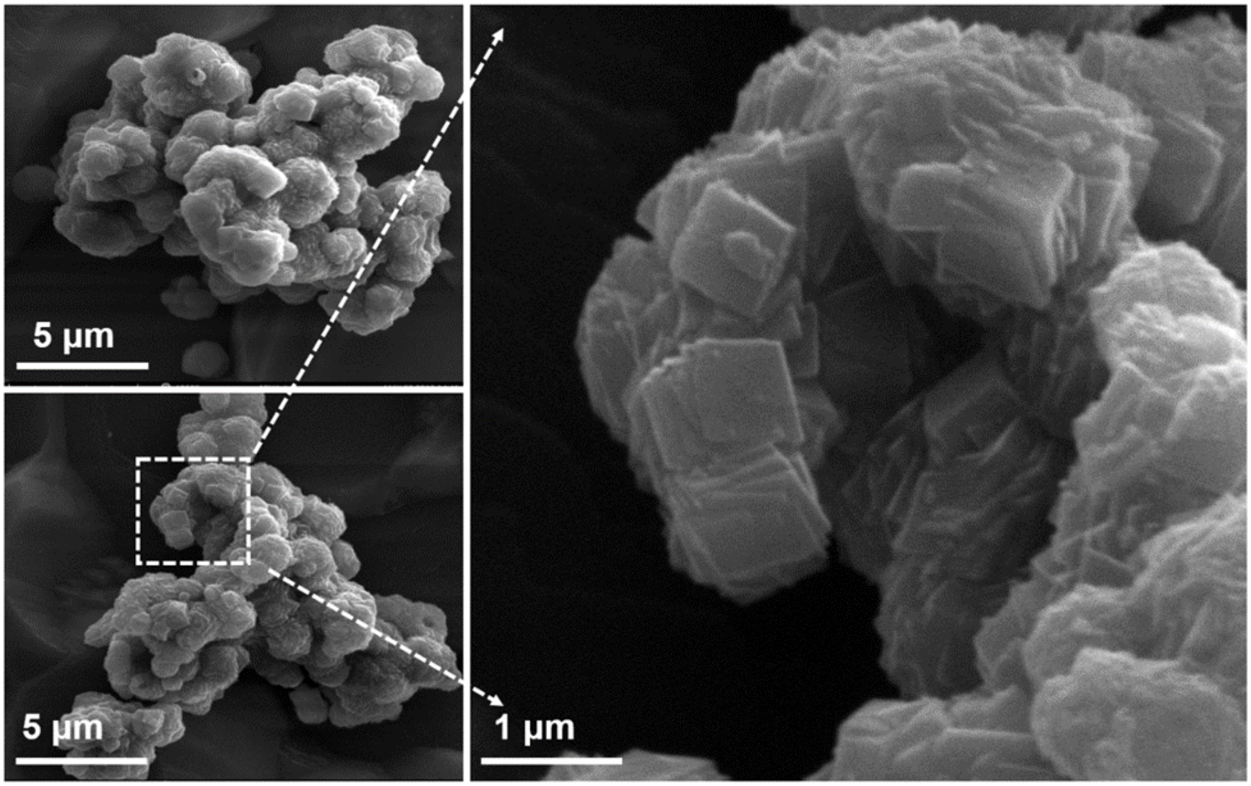

Supplementary Figure 5. The scanning electron microscopy (SEM) micrographs of pristine Li_2MnO_3.

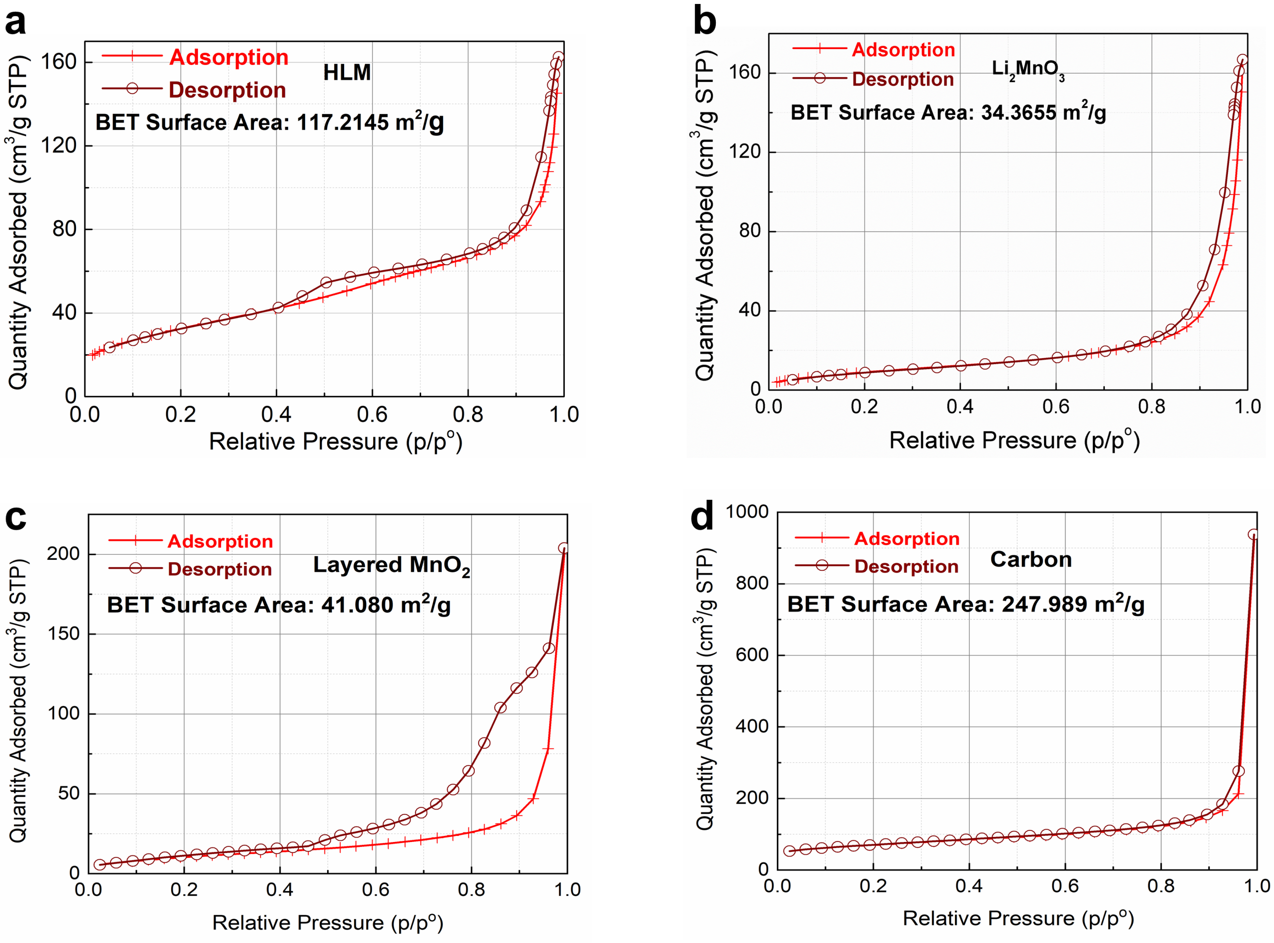

Supplementary Figure 6. N_2 adsorption/desorption isotherms of HLM, Li_2MnO_3, Layered MnO_2 and carbon.

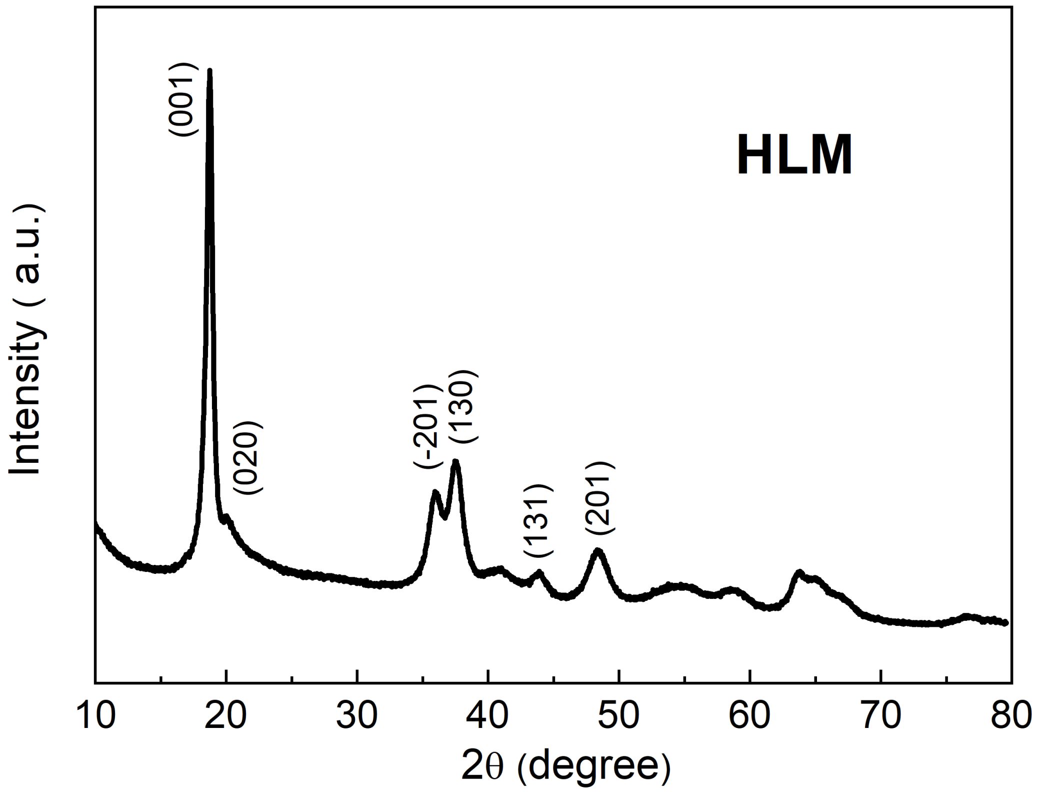

Supplementary Figure 7. XRD pattern with Miller indices (hkl) for HLM.

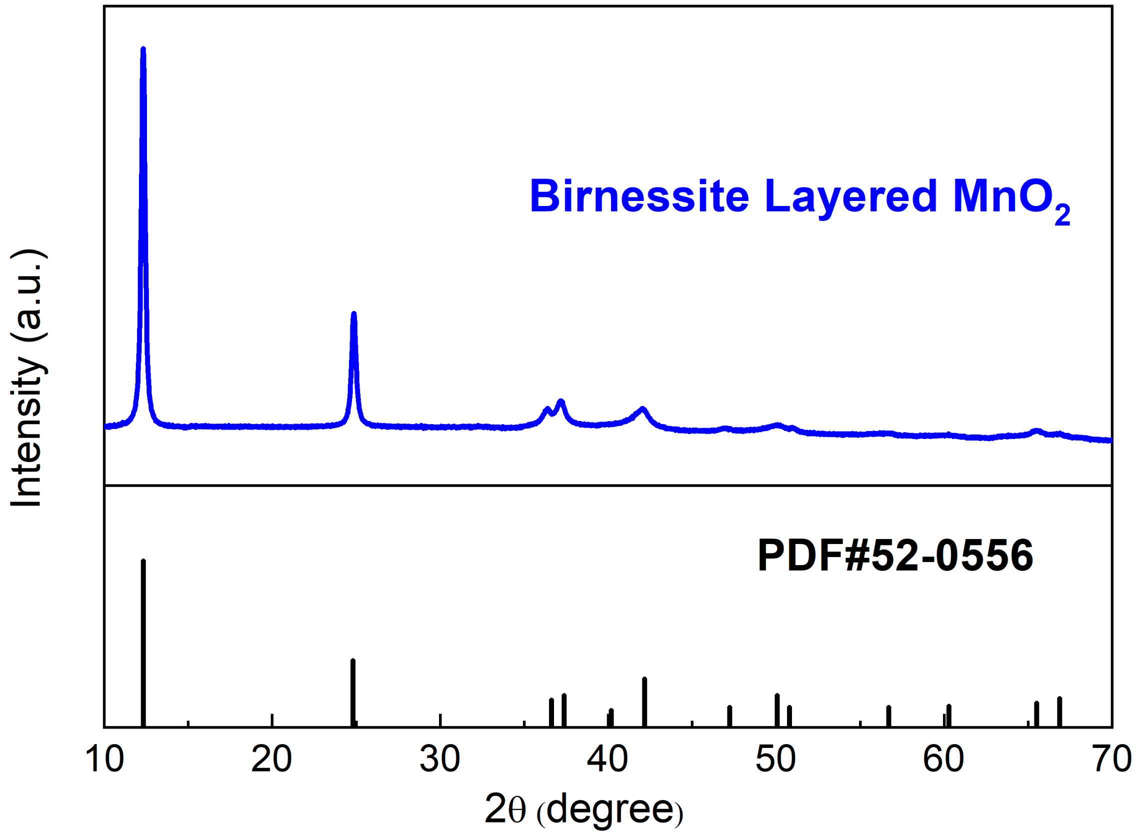

Supplementary Figure 8. XRD pattern for Birnessite Layered MnO_2.

Supplementary Section 2. Electrochemical measurements.

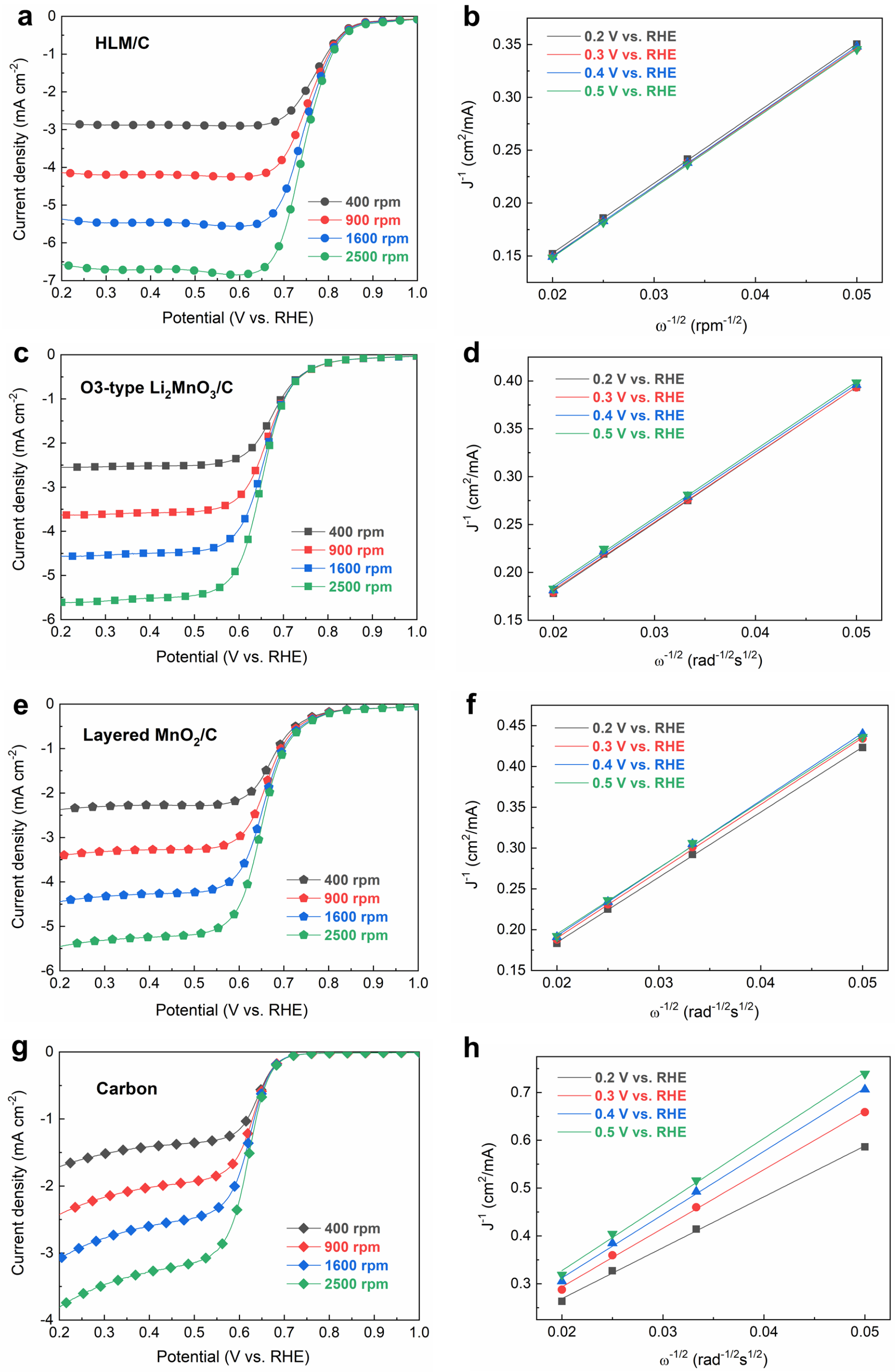

Supplementary Figure 9. Linear sweeping voltammograms curves and corresponding Koutecky-Levich plots of (**a**, **b**) HLM/C, (**c**, **d**) Li_2MnO_3/C, (**e**, **f**) layered MnO_2/C, (**g**, **h**) VulcanXC-72R obtained at O_2-saturated 0.1 M KOH with a scan rate of 5 mV s^{-1}.

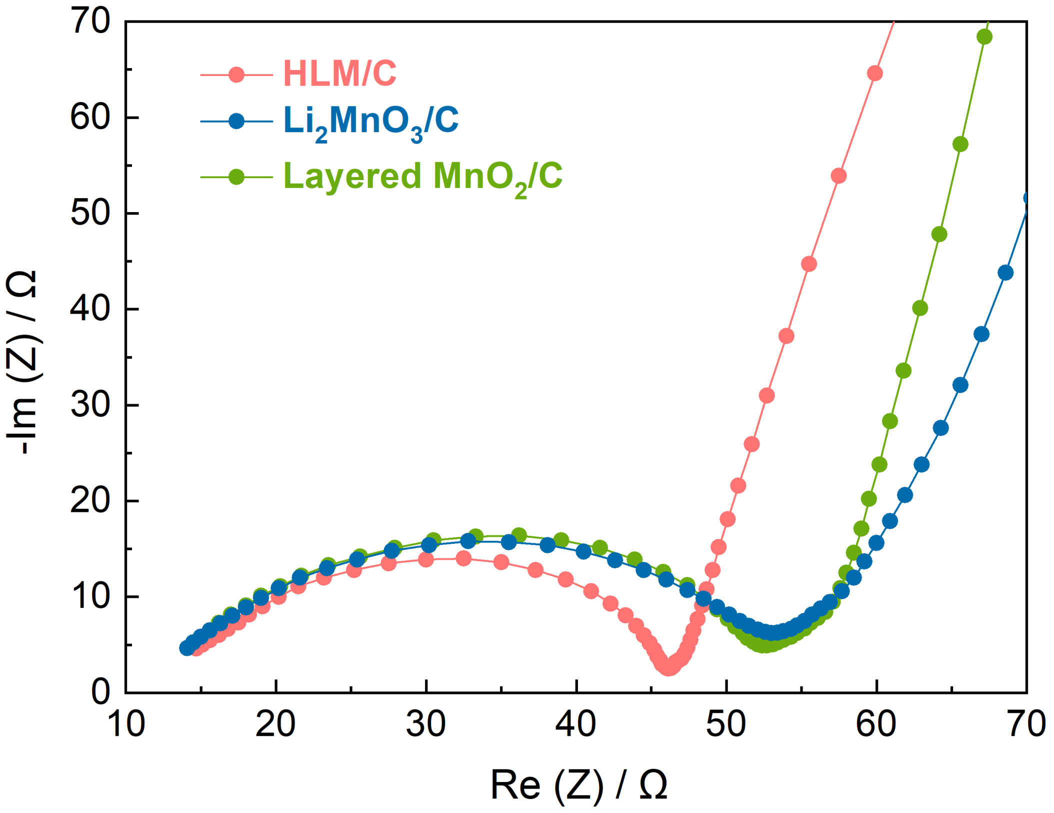

Supplementary Figure 10. Nyquist plots studied by using EIS measurement in 0.1M KOH solution saturated with O_2.

Supplementary Table 2. Summary of reported state-of-the-art Mn-based oxides and noble metal catalysts in terms of onset potentials and half-wave potentials in alkaline conditions.

Catalysts	Mass loading (mg cm^{-2})	Onset potentials (V vs. RHE)	Half-wave potentials (V vs. RHE)	Ref
HLM	~0.12	0.95	0.75	This work
Commercial MnO_2	~0.08	0.66	0.58	1
MnO_2 nanosheets	~0.08	0.79	0.70	1
MnO	-	0.69	0.58	2
δ-MnO_2	0.204	0.71	0.66	3
β-MnO_2	0.204	0.86	0.69	3
MnO_2 nanoflowers	0.255	0.81	0.64	4
20 wt% Pt/C	0.255	0.93	0.8	4
MnO_2 nanorod	0.465	0.73	0.61	5
Mn_3O_4	0.31	0.74	0.57	6
Tremella-like δ-MnO_2	0.076	0.82	0.74	7
Ag-MnO_2	0.063	0.83	0.67	8
N-Carbon/MnO_2	0.085	0.88	0.65	9
Porous MnO_2	-	0.84	0.62	10
Mn_2O_3	0.044	0.80	0.60	11
α-MnO_2	0.25	0.82	0.71	12
Ar-350-2h-MnO_2	0.073	0.87	0.72	13
λ-MnO_2	0.17	0.85	0.68	14
20wt% Ru/C	-	0.81	0.62	15
6 wt% Pt/C	0.74	0.92	0.73	16
H-MnO_2/C	0.089	0.96	0.76	17

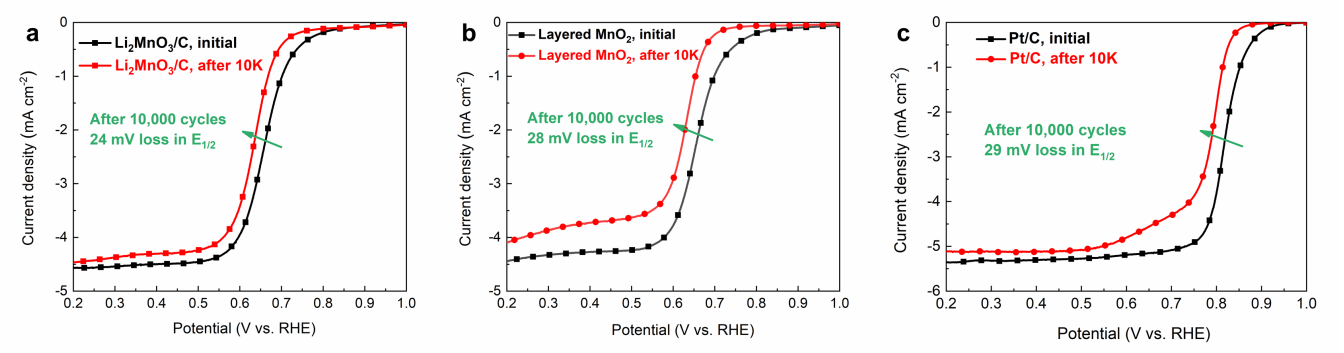

Supplementary Figure 11. Catalyst stability studied by using potential cycling (from 0.6 V to 1 V vs. RHE) of (**a**) Li_2MnO_3/C, (**b**) layered MnO_2/C and (**c**) 20 wt% Pt/C (Johnson-Matthey), at a scan rate of 100 mv s^{-1} in O_2-saturated 0.1M KOH.

Supplementary Section 3. Summary of non-noble metal catalysts in terms of ORR stability.

Supplementary Table 3. Summary of reported non-noble metal and Pt-based catalysts in terms of ORR stability in alkaline conditions.

Catalysts	Stability, $E_{1/2}$	Diffusion-limiting current density (1600 rpm)	ref
HLM/C	-10 mV (10000 cycles)	-5.46 mA/cm^2	This work
MnO_2/C	-76 mV (3000 cycles)	-4.1 mA/cm^2	18
GNF-L/N-F	-30 mV (10000 cycles)	-4.5 mA/cm^2	19
Pt/C	-50 mV (1000 cycles)	--	19
GNF-A/N-F	-20 mV (10000 cycles)	-5.0 mA/cm^2	19
Fe–N/C-120	I/I0=92% (E=0.4 V, 2.78 h)	--	20
FeN_2/NOMC-3	I/I0=93% (E=0.79 V, 0.83 h)	-5.2 mA/cm^2	21
Au–Pt aerogel	-12 mV (1000 cycles)	-6.1 mA/cm^2	22
Au–Pt NPs	-30 mV (1000 cycles)	-5.1 mA/cm^2	22
Fe-N-C HNSs	I/I0=91% (E=0.7 V, 5.55 h)	-5.9 mA/cm^2	23
CoNMC-700-1	-16.5 mV (2000 cycles)	-6.42 mA/cm^2	24
Pt/C	-47 mV (2000 cycles)	-6.1 mA/cm^2	24
10Co-N@DCNF	-10 mV (5000 cycles)	-6.36 mA/cm^2	25
Fe-N/GNs	-19 mV (4000 cycles)	-5.24 mA/cm^2	26
3DOM Fe–N–C-900	-2 mV (10000 cycles)	-6.1 mA/cm^2	27
Ni_3Fe/N-C	I/I0=96.5% (E=0.7 V, 3.33 h)	-6.0 mA/cm^2	28
P-Fe-NC	Less than 10 mV (10000 cycles)	-5.0 mA/cm^2	29
FeNC-950	-11 mV (10000 cycles)	-5.85 mA/cm^2	30
IR/CN-50%	I/I0=83.5% (E=0.4 V, 8.33 h)	-6.35 mA/cm^2	31
2DPCs-a	I/I0=80.2% (E=0.75 V, 9.72 h)	-5.2 mA/cm^2	32

Supplementary Section 4. Mechanistic study.

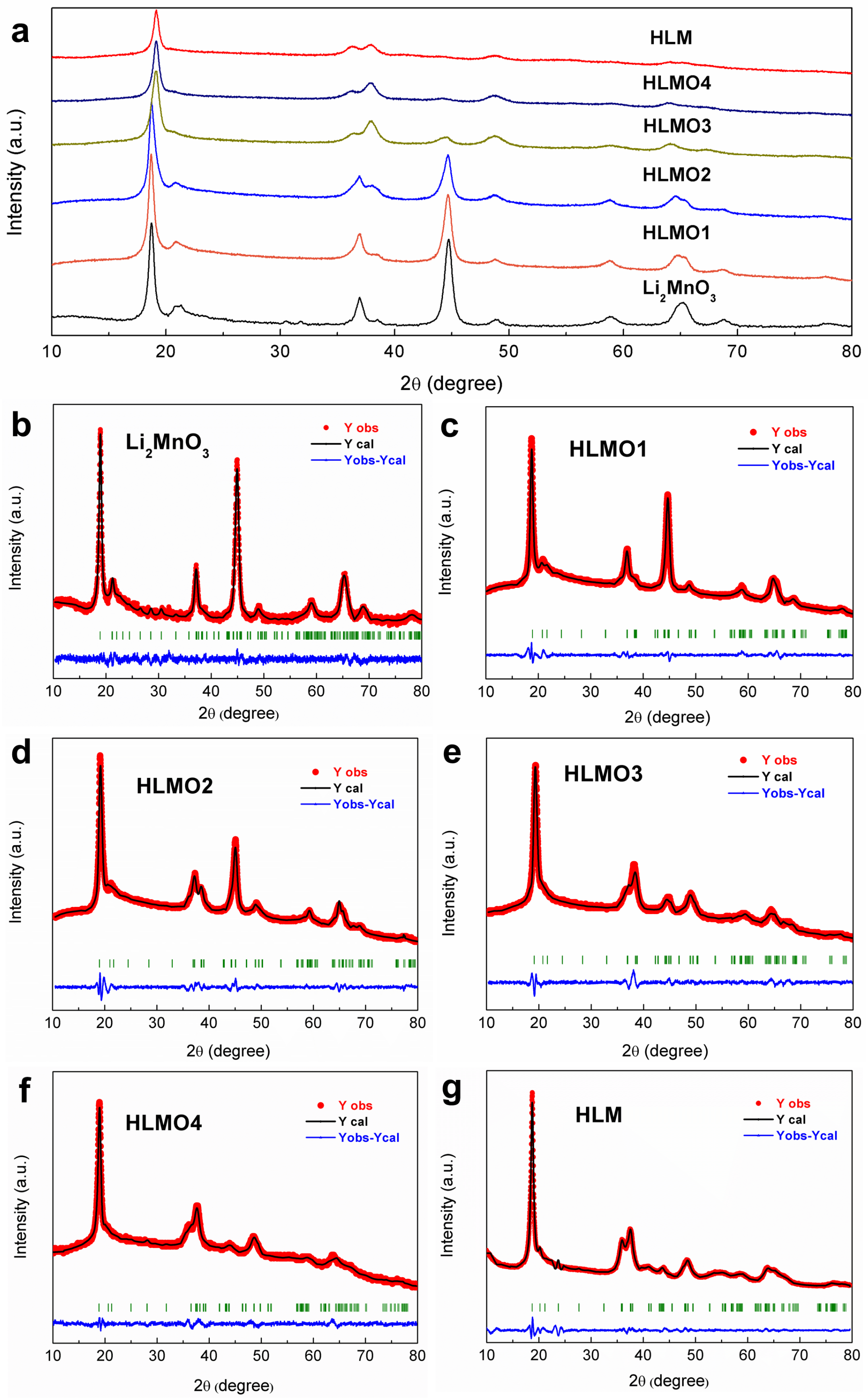

Supplementary Figure 12. XRD patterns and corresponding Rietveld refinement profiles of Li_2MnO_3 and protonated samples.

Supplementary Table 4. Refined crystal structural data for Li_2MnO_3 and protonated samples.

Compounds	Li/Mn ratio (ICP-OES)	*a* (Å)	*b* (Å)	*c* (Å)	*β* (°)	*V* (Å^3)	$d_{O\text{-}O}$ (Å) (Interlayer distance)
Li_2MnO_3	1.99	4.918	8.487	4.945	108.8	195.3	3.03
HLMO1	1.38	4.932	8.490	4.929	108.7	195.6	2.86
HLMO2	0.97	4.943	8.510	4.919	108.8	195.8	2.84
HLMO3	0.42	4.965	8.539	4.901	109.0	196.5	2.69
HLMO4	0.29	4.969	8.559	4.899	109.1	196.8	2.60
HLM	0.25	5.016	8.622	4.858	107.7	200.2	2.53

Supplementary Table 5. Fit goodness and R-factor of Rietveld refinements for Li_2MnO_3 and protonated samples.

Samples	χ^2	R_F	R_B	R_p	R_{wp}
Li_2MnO_3	1.61	1.36%	2.59%	18.7%	17.7%
HLMO1	1.44	4.46%	1.59%	26.1%	12.7%
HLMO2	1.65	1.02%	1.00%	29.4%	16.8%
HLMO3	1.10	4.20%	1.30%	20.6%	16.7%
HLMO4	1.22	1.69%	1.16%	21.7%	20.1%
HLM	1.50	2.1%	4.3%	19.1%	17.4%

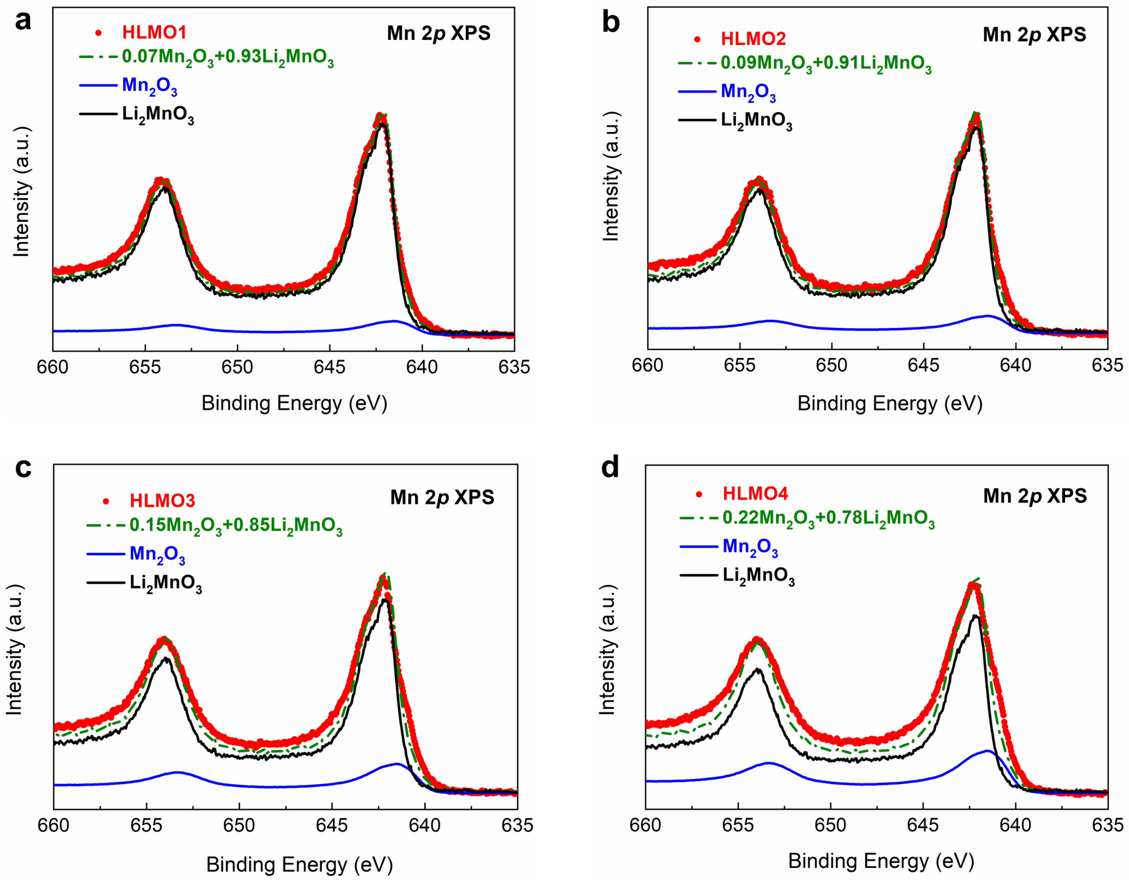

Supplementary Figure 13. High-resolution Mn 2*p* XPS spectra of protonated samples.

Supplementary Table 6. Mn $2p_{3/2}$ XPS parameters for protonated samples.

Catalysts	Ratio of Li/Mn (ICP-OES)	Mn^{3+}/Mn^{4+} Reference	Percentage (%)	Average valence state of Mn
HLMO1	1.38	Mn_2O_3	7	3.93
		Li_2MnO_3	93	
HLMO2	0.97	Mn_2O_3	9	3.91
		Li_2MnO_3	91	
HLMO3	0.42	Mn_2O_3	15	3.85
		Li_2MnO_3	85	
HLMO4	0.29	Mn_2O_3	22	3.78
		Li_2MnO_3	78	
HLM	0.25	Mn_2O_3	30	3.70
		Li_2MnO_3	70	

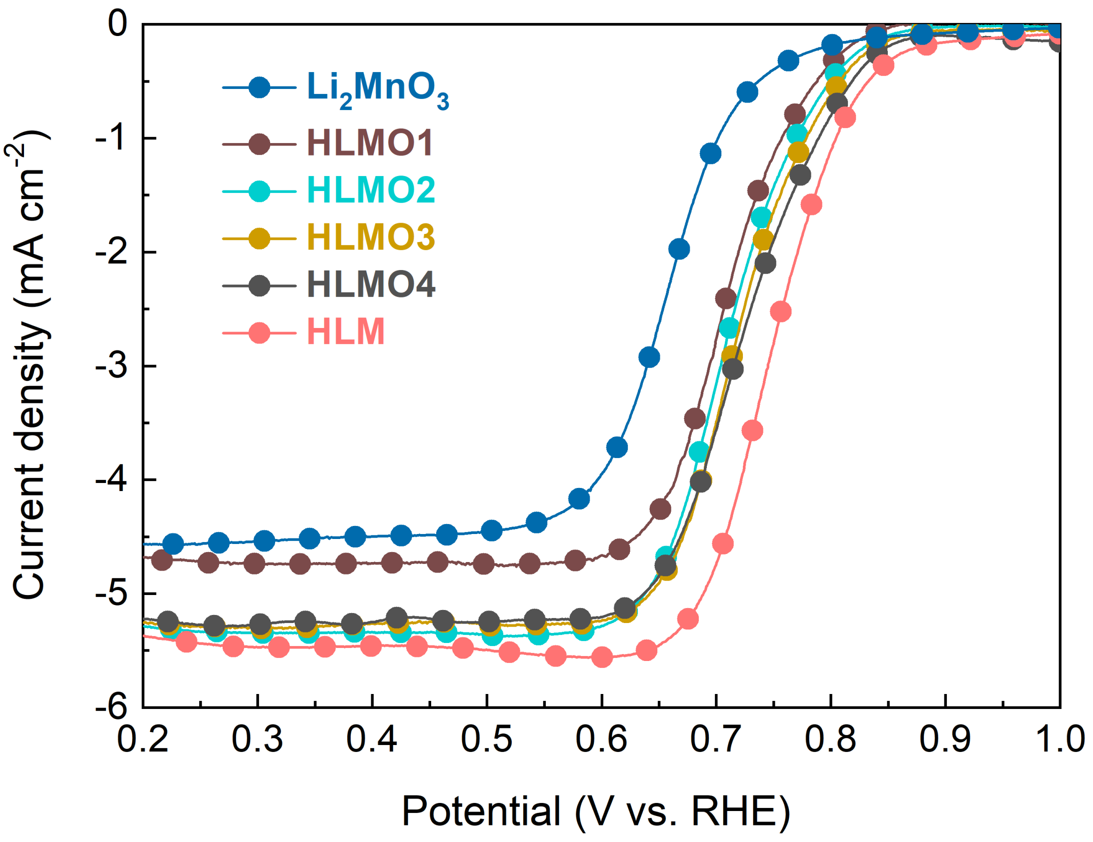

Supplementary Figure 14. ORR polarization curve of Li_2MnO_3 and protonated samples at a rotating speed of 1600 rpm in O_2-saturated 0.1 M KOH solution.

Supplementary Section 5. Micro Laminar Flow Fuel Cell performance comparison.

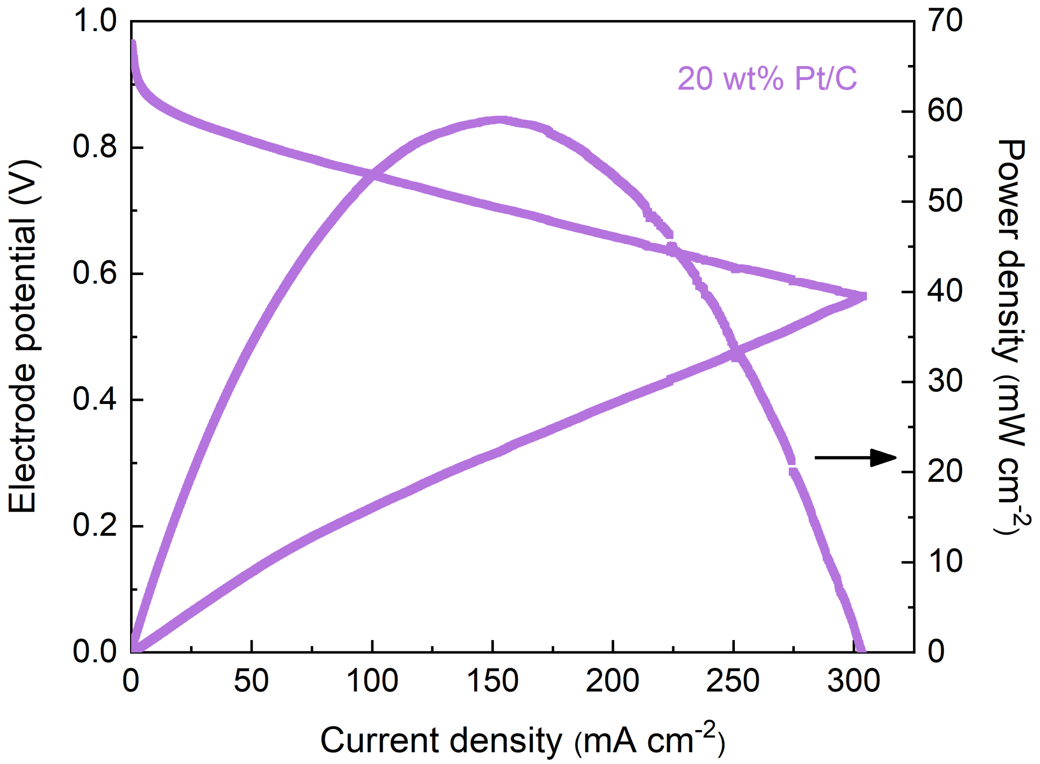

Supplementary Figure 15. Current-Potential characteristics and corresponding power density curves of 20 wt% Pt/C in the H_2/O_2-μLFFCs.

Supplementary Table 7. Comparison of the HLM with other reported catalysts in the alkaline-based micro fuel cells, in terms of current density and power density.

Micro-fuel cell design	Cathode-Anode	Fuel/oxidant	[a] j_{max}/mA cm^{-2} or Current density @ P_{max} mA/cm^2	[a] P_{max} /mW cm^{-2}	Ref
H_2/O_2 micro-fuel cell	HLM/C-Pt/C	H_2/O_2+3M KOH	229.05 mA cm^{-2} (j_{max}/mA cm^{-2})	40.01	This work
H_2/O_2 micro-fuel cell	Pt/C-Pt/C	H_2/O_2+3M KOH	~305.7 mA cm^{-2} (j_{max}/mA cm^{-2})	59.2	33
H_2/O_2 micro-fuel cell	NC25(1000)-Pt/C	H_2/O_2+3M KOH	~215.7 mA cm^{-2} (j_{max}/mA cm^{-2})	39.9	33
H_2/O_2 micro-fuel cell	CoSe/C-Pt/C	H_2/O_2+3M KOH	~226 mA cm^{-2} (j_{max}/mA cm^{-2})	42.7	33
Alkaline membrane fuel cell	#3α-MnO_2/C (1 mg cm^{-2})-Pt/C	H_2/O_2+1M KOH (50 °C)	-	35.3	34
Alkaline membrane fuel cell	#5α-MnO_2/C (2 mg cm^{-2})-Pt/C	H_2/O_2+1M KOH (50 °C)	-	23.8	34
Alkaline membrane fuel cell	#8α-MnO_2/C (2 mg cm^{-2})-Pt/C	H_2/O_2+1M KOH (50 °C)	-	17.3	34
Direct glucose alkaline fuel cell	Au/MnO2/C (3 mg cm^{-2})-Activated Charcoal	0.3 M Glucose+1 M KOH	2.5 mA cm^{-2} (Current density @ P_{max} mA/cm^2)	1.1	35
Microfluidic H_2/O_2 fuel cell	Pt/C (280 cycle, 2 mg cm^{-2})-Pt/C	H_2/O_2+1M KOH	-	11.4	36
Microfluidic H_2/O_2 fuel cell	Pt/C (410 cycle, 2 mg cm^{-2})-Pt/C	H_2/O_2+1M KOH	-	30.4	36
Flowing alkaline electrolyte fuel cell	MnO_2 (3 mg cm^{-2})-Pt/Ni (Pt loading of 1.2 mg cm^{-2})	1M Methanol +3M KOH	28.5 mA cm^{-2} (Current density @ P_{max} mA/cm^2)	9.2	37
Flowing alkaline electrolyte fuel cell	MnO_2 (3 mg cm^{-2})-Pt/Ni (1.2 mg cm^{-2} Pt loading)	1M Sodium borohydride +3M KOH	39 mA cm^{-2} (Current density @ P_{max} mA/cm^2)	19	37
Flowing alkaline electrolyte fuel cell	MnO_2 (3 mg cm^{-2})-Pt/Ni (1.2 mg cm^{-2} Pt loading)	1M Ethanol +3M KOH	34 mA cm^{-2} (Current density @ P_{max} mA/cm^2)	14.6	37
Polymer Electrolyte Membrane Fuel Cells	Au–MnO_2/ MWNT (1 mg cm^{-2})-Pt/MWNT (Pt loading of 0.25 mg cm^{-2})	-	~63 mA cm^{-2} (Current density @ P_{max} mA/cm^2)	~32	38
Microfluidic hydrogen–oxygen (H_2/O_2) fuel cell	Ag/C (4mg Ag cm^{-2})-Pt/C (1mg Pt cm^{-2})	H_2/O_2+9M KOH	~114 mA cm^{-2} (jmax/mA cm^{-2})	25	39
Microfluidic hydrogen–oxygen (H_2/O_2) fuel cell	Ag/C (4mg Ag cm^{-2})-Pt/C (1mg Pt cm^{-2})	H_2/O_2+7M KOH	~189 mA cm^{-2} (jmax/mA cm^{-2})	29	39
Microfluidic hydrogen–oxygen (H_2/O_2) fuel cell	Ag/C (4mg Ag cm^{-2})-Pt/C (1mg Pt cm^{-2})	H_2/O_2+5M KOH	~266 mA cm^{-2} (jmax/mA cm^{-2})	39	39
Tolerant MeOH chalcogenides cathodes μFC	$CoSe_2$/NCNH-PtRu/C	MeOH + KOH/O_2 + KOH	124.2 mA cm^{-2} (jmax/mA cm^{-2})	10.04	40
H_2O_2 dual electrolyte μFC	No data	H_2O_2 + NaOH/ H_2O_2 + H_2SO_4	-	24	41

(a) The onset potential, current density, and power density were obtained from the text or read from the graphics.

Supplementary Note 1

Synthesis.

Synthesis of precursor O3-type Li_2MnO_3. The precursor O3-type Li_2MnO_3 was prepared by a solid-state reaction. Briefly, Mn_2CO_3 (≥99.9%, Aladdin) and LiOH (≥99.9%, Alfa Aesar) with a molar ratio of 1:2 was ground in a planet-type ball mill for 12 hours, transferred into the muffle furnace and heated at 450 ˚C for 40 h in the air to obtain Li_2MnO_3.

Synthesis of P3-type $H_{1.0}(H_{0.13}Li_{0.17}\square_{0.03}Mn_{0.67})O_{1.89}\square_{0.11}$ (HLM). The preparation of layered P3-type defective protonated lithium manganese oxide follows a facile treatment using acidic leaching. Briefly, a solution containing 0.075 mol H_2SO_4 (93-98%, Alfa Aesar) and 30 ml of deionized water was added to 300 mg of the obtained brown pristine Li_2MnO_3 sample to prepare HLM sample. The solution was stirred at 25 ˚C for 12 h. The precipitate was collected by centrifugation and washed with deionized water 3 times and dried overnight at 60 ˚C under vacuum.

Synthesis of Layered MnO_2 (δ-MnO_2, Birnessite). The layered MnO_2 was prepared by the following process: 10 mmol of $KMnO_4$ (≥99.0%, Alfa Aesar) was dissolved in 65 ml of deionized water and stir for 10 min. The solution was then transferred to Teflon-lined autoclave with a filling rate of 70%, and kept in an oven at 220 ˚C for 48 h. After cooling, the resulting products were centrifuged, washed with 120 ml of deionized water 4 times, and then dried in the oven at 60 ˚C.

Synthesis of different protonated samples. A series of protonated samples were prepared by acid treatment on Li_2MnO_3 by varying acid concentrations. The samples were delithiated by adding the solutions containing 0.642 mmol, 1.284 mmol, 1.926 mmol and 2.568 mmol H_2SO_4 (93-98%, Alfa Aesar) and 30 ml of deionized water to 300 mg of Li_2MO_3 sample, respectively. The solution was stirred at 25 ˚C for 12 h. The obtained samples are denoted as HLMO1, HLMO2, HLMO3 and HLMO4, respectively. The number of extracted Li were calculated based on the ratios of Li/Mn in HLMO1, HLMO2, HLMO3 and HLMO4, determined by ICP-OES.

Supplementary Note 2

Discussion on *U* values in DFT+*U* calculations.

We used DFT+*U* method to perform our first-principles calculations, with *U* value added as on-site penalty energy on the Mn *d* orbital to incorporate its strong correlation. However, one has to note that the real physical system with multiple ions and electrons is actually a many-body problem, while DFT+*U* scheme is still in the framework of single electron approximation. In practice, one usually only selects a typical *U* value in a reasonable energy range (e.g., usually 3 – 6 eV for Mn *d* orbital) to perform the DFT+*U* calculations. If the calculated results show qualitatively unchanged behavior in this energy range, then such calculations are generally reliable. Indeed, Tamura el al.[42] have compared the geometric structure and electronic states of Li_2MnO_3 with *U* values taken in the range of 3 – 7 eV, which show qualitatively same results. Hence, in our calculations we adopt U = 4 eV, which should reflect the correct main electronic behavior of both Li_2MnO_3 and HLM. One has to note that calculations based on the many-body theory (configuration interaction (CI) calculations, as performed in the current work, but not for large systems) could yield benchmark results to guide DFT+*U*. In this work, the CI calculations, experimental observations, and DFT+*U* results show good consistency.

In addition, we studied the influence of *U* corrections for O 2*p* states when O vacancies were presented in the HLM system. Indeed, exact evaluations of thermodynamic and electronic properties of such point defects are complicated in DFT-based calculations, which incorporates 3D periodic boundary condition. A possible method is to apply a finite *U* on-site energy correction on the O 2*p* states, which may help its convergence of electron spectrum and yield good real space localization, especially when the calculations are performed in the small simulation supercells. We performed additional calculations by adding *U* corrections on O 2*p* orbitals (denoted by U_O) in the HLM system, from U_O = 0 eV to U_O = 6 eV. The main feature of the electron spectra does not change (Supplementary **Figure 16**), indicating our defective HLM system is not sensitive to the *U* corrections of O 2*p* orbitals. Therefore, most of the caveats from standard GGA methods can be corrected by DFT+*U* (*U* for Mn *d* orbitals). Moreover, Ricca et al.[43] compared the DFT+*U* and the state-of-the-art DFT+*U*+*V* (*V* represents the strength of the interaction between electrons on neighbor O sites, which could yield good results comparable with when adopting hybrid functional)

performances on oxygen vacancies in $SrTiO_3$, indicating that the DFT+U (U for d orbitals) calculations could give qualitatively correct results as compared with the DFT+U+V calculations. Moreover, our calculations yield consistent results with experimental observations, when adopting DFT+U calculation with on-site energy correction for only Mn d orbitals.

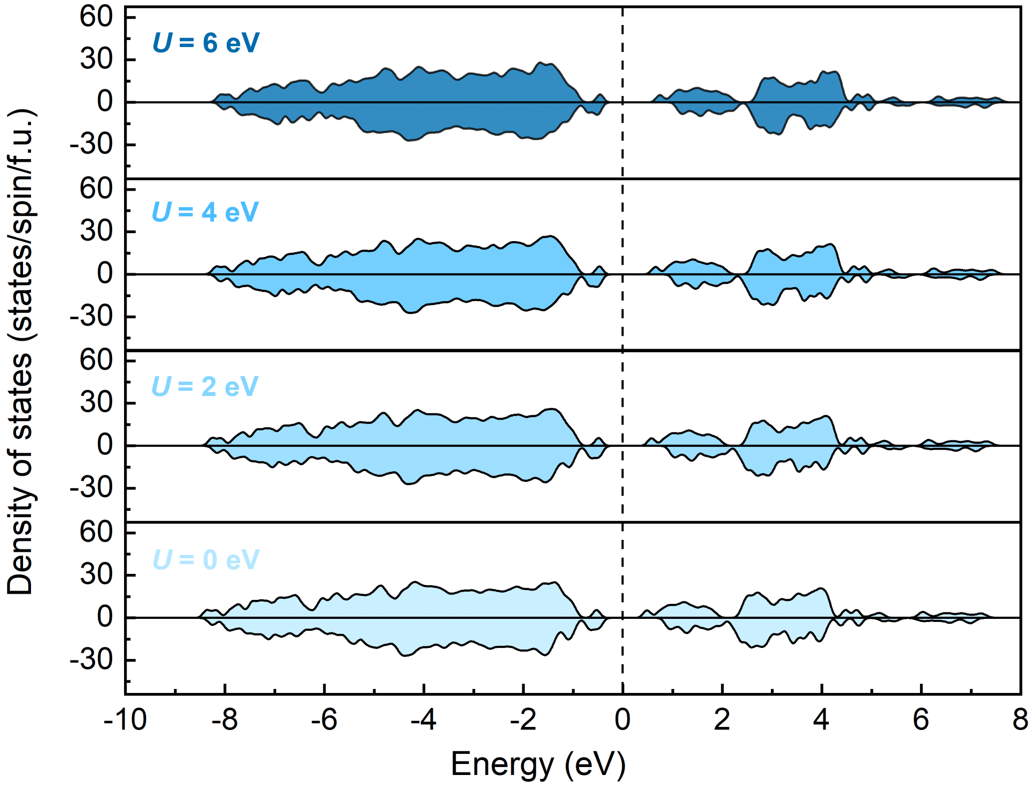

Supplementary Figure 16. Density of states calculated for the HLM system with different U_O corrections on O 2p orbital. The U correction of 4 eV on Mn d orbital is always applied.

Supplementary Reference

1 Shi, C., Zang, G.-L., Zhang, Z., Sheng, G.-P., Huang, Y.-X., Zhao, G.-X., Wang, X.-K. & Yu, H.-Q. Synthesis of layered MnO_2 nanosheets for enhanced oxygen reduction reaction catalytic activity. *Electrochim. Acta* **132**, 239-243 (2014).

2 Chen, R., Yan, J., Liu, Y. & Li, J. Three-dimensional nitrogen-doped graphene/MnO nanoparticle hybrids as a high-performance catalyst for oxygen reduction reaction. *J. Phys. Chem. C* **119**, 8032-8037 (2015).

3 Meng, Y., Song, W., Huang, H., Ren, Z., Chen, S.-Y. & Suib, S. L. Structure–property relationship of bifunctional MnO_2 nanostructures: highly efficient, ultra-stable electrochemical water oxidation and oxygen reduction reaction catalysts identified in alkaline media. *J. Am. Chem. Soc.* **136**, 11452-11464 (2014).

4 Yang, J., Wang, J., Zhu, L., Gao, Q., Zeng, W., Wang, J. & Li, Y. Enhanced electrocatalytic activity of a hierarchical CeO_2@ MnO_2 core-shell composite for oxygen reduction reaction. *Ceram. Int.* **44**, 23073-23079 (2018).

5 Yue, P., Li, Z., Wang, S. & Wang, Y. MnO_2 nanorod catalysts for magnesium–air fuel cells: Influence of different supports. *Int. J. Hydrogen Energy.* **40**, 6809-6817 (2015).

6 Lv, X., Lv, W., Wei, W., Zheng, X., Zhang, C., Zhi, L. & Yang, Q.-H. A hybrid of holey graphene and Mn_3O_4 and its oxygen reduction reaction performance. *Chem. Commun.* **51**, 3911-3914 (2015).

7 Ma, Y., Wang, R., Wang, H., Key, J. & Ji, S. Control of MnO_2 nanocrystal shape from tremella to nanobelt for ehancement of the oxygen reduction reaction activity. *J. Power Sources* **280**, 526-532 (2015).

8 Sun, S., Miao, H., Xue, Y., Wang, Q., Li, S. & Liu, Z. Oxygen reduction reaction catalysts of manganese oxide decorated by silver nanoparticles for aluminum-air batteries. *Electrochim. Acta* **214**, 49-55 (2016).

9 Li, Y., Cao, S., Fan, L., Han, J., Wang, M. & Guo, R. Hybrid shells of MnO_2 nanosheets encapsulated by N-doped carbon towards nonprecious oxygen reduction reaction catalysts. *J. Colloid Interface Sci.* **527**, 241-250 (2018).

10 Zuo, L.-X., Jiang, L.-P., Abdel-Halim, E. S. & Zhu, J.-J. Sonochemical preparation of stable porous MnO_2 and its application as an efficient electrocatalyst for oxygen reduction reaction. *Ultrason. Sonochem.* **35**, 219-225 (2017).

11 Cao, S., Han, N., Han, J., Hu, Y., Fan, L., Zhou, C. & Guo, R. Mesoporous hybrid shells of carbonized polyaniline/Mn_2O_3 as non-precious efficient oxygen reduction reaction catalyst. *ACS Appl. Mater. Interfaces* **8**, 6040-6050 (2016).

12 Shi, X., Ahmad, S., Pérez-Salcedo, K., Escobar, B., Zheng, H. & Kannan, A. M. Maximization of quadruple phase boundary for alkaline membrane fuel cell using non-stoichiometric α-MnO_2 as cathode catalyst. *Int. J. Hydrogen Energy* **44**, 1166-1173 (2019).

13 Cheng, F., Zhang, T., Zhang, Y., Du, J., Han, X. & Chen, J. Enhancing electrocatalytic oxygen reduction on MnO_2 with vacancies. *Angew. Chem. Int. Ed.* **52**, 2474-2477 (2013).

14 Lee, S., Nam, G., Sun, J., Lee, J.-S., Lee, H.-W., Chen, W., Cho, J. & Cui, Y. Enhanced Intrinsic Catalytic Activity of λ-MnO_2 by Electrochemical Tuning and Oxygen Vacancy Generation. *Angew. Chem. Int. Ed.* **55**, 8599-8604 (2016).

15 Pickrahn, K. L., Park, S. W., Gorlin, Y., Lee, H.-B.-R., Jaramillo, T. F. & Bent, S. F. Active MnO_x electrocatalysts prepared by atomic layer deposition for oxygen evolution and oxygen reduction reactions. *Adv. Energy Mater.* **2**, 1269-1277 (2012).

16 Zhang, Z., Li, Z., Sun, C., Zhang, T. & Wang, S. Preparation and properties of an amorphous MnO_2/CNTs-OH catalyst with high dispersion and durability for magnesium-air fuel cells. *Catal. Today* **298**, 241-249 (2017).

17 Zhang, T., Cheng, F., Du, J., Hu, Y. & Chen, J. Efficiently enhancing oxygen reduction electrocatalytic activity of MnO_2 using facile hydrogenation. *Adv. Energy Mater.* **5**, 1400654 (2015).

18 Li, L., Feng, X., Nie, Y., Chen, S., Shi, F., Xiong, K., Ding, W., Qi, X., Hu, J., Wei, Z., Wan, L.-J. & Xia, M. Insight into the effect of oxygen vacancy concentration on the catalytic performance of MnO_2. *ACS Catal.* **5**, 4825-4832 (2015).

19 Akula, S. & Sahu, A. K. Structurally Modulated Graphitic Carbon Nanofiber and Heteroatom (N,

F) Engineering toward Metal-Free ORR Electrocatalysts for Polymer Electrolyte Membrane Fuel Cells. *ACS Appl. Mater. Interfaces.* **12**, 11438-11449 (2020).

20 Zhong, W., Chen, J., Zhang, P., Deng, L., Yao, L., Ren, X., Li, Y., Mi, H. & Sun, L. Air plasma etching towards rich active sites in Fe/N-porous carbon for the oxygen reduction reaction with superior catalytic performance. *J. Mater. Chem. A.* **5**, 16605-16610 (2017).

21 Shen, H., Gracia-Espino, E., Ma, J., Tang, H., Mamat, X., Wagberg, T., Hu, G. & Guo, S. Atomically FeN_2 moieties dispersed on mesoporous carbon: A new atomic catalyst for efficient oxygen reduction catalysis. *Nano Energy* **35**, 9-16 (2017).

22 Du, R., Jin, W., Hübner, R., Zhou, L., Hu, Y. & Eychmüller, A. Engineering Multimetallic Aerogels for pH-Universal HER and ORR Electrocatalysis. *Adv. Energy Mater.* **10**, 1903857 (2020).

23 Chen, Y., Li, Z., Zhu, Y., Sun, D., Liu, X., Xu, L. & Tang, Y. Atomic Fe Dispersed on N-Doped Carbon Hollow Nanospheres for High-Efficiency Electrocatalytic Oxygen Reduction. *Adv. Mater.* **31**, 1806312 (2019).

24 Rong, Z., Dong, C., Zhang, S., Dong, W. & Huang, F. $Co_{5.47}N$ loaded N-doped carbon as an efficient bifunctional oxygen electrocatalyst for a Zn–air battery. *Nanoscale* **12**, 6089-6095 (2020).

25 Yang, Q., Jia, Y., Wei, F., Zhuang, L., Yang, D., Liu, J., Wang, X., Lin, S., Yuan, P. & Yao, X. Understanding the Activity of $Co\text{-}N_{4-x}C_x$ in Atomic Metal Catalysts for Oxygen Reduction Catalysis. *Angew. Chem.* **132**, 6178-6183 (2020).

26 Liu, D., Li, J.-C., Ding, S., Lyu, Z., Feng, S., Tian, H., Huyan, C., Xu, M., Li, T., Du, D., Liu, P., Shao, M. & Lin, Y. 2D Single-Atom Catalyst with Optimized Iron Sites Produced by Thermal Melting of Metal–Organic Frameworks for Oxygen Reduction Reaction. *Small Methods* **4**, 1900827 (2020).

27 Zhang, X., Han, X., Jiang, Z., Xu, J., Chen, L., Xue, Y., Nie, A., Xie, Z., Kuang, Q. & Zheng, L. Atomically dispersed hierarchically ordered porous Fe–N–C electrocatalyst for high performance electrocatalytic oxygen reduction in Zn-Air battery. *Nano Energy* **71**, 104547 (2020).

28 Fu, G., Cui, Z., Chen, Y., Li, Y., Tang, Y. & Goodenough, J. B. Ni_3Fe-N doped carbon sheets as a bifunctional electrocatalyst for air cathodes. *Adv. Energy Mater.* **7**, 1601172 (2017).

29 Deng, J., Chen, S., Zhou, Q., Nie, Y., Li, J., Wu, R., Wang, Q. & Wei, Z. Phytic acid-assisted self-templating synthesis of NP-Fe-tridoped hierarchical porous carbon for efficient oxygen reduction reaction. *J. Power Sources* **451**, 227808 (2020).

30 Ma, Y., Luo, S., Tian, M., Lu, J. E., Peng, Y., Desmond, C., Liu, Q., Li, Q., Min, Y., Xu, Q. & Chen, S. Hollow carbon spheres codoped with nitrogen and iron as effective electrocatalysts for oxygen reduction reaction. *J. Power Sources* **450**, 227659 (2020).

31 Wang, Y., Zhong, K., Huang, Z., Chen, L., Dai, Y., Zhang, H., Su, M., Yan, J., Yang, S., Li, M., Xu, T. & Tang, J. Novel g-C_3N_4 assisted metal organic frameworks derived high efficiency oxygen reduction catalyst in microbial fuel cells. *J. Power Sources* **450**, 227681 (2020).

32 Tu, K., Zou, L., Yang, C., Su, Y., Lu, C., Zhu, J., Zhang, F., Ke, C. & Zhuang, X. Ionic Polyimide Derived Porous Carbon Nanosheets as High–Efficiency Oxygen Reduction Catalysts for Zn–Air Batteries. *Chem. Eur. J.* **26**, 1 – 11 (2020).

33 García-Rosado, I. J., Uribe-Calderon, J. & Alonso-Vante, N. Nitrogen-doped reduced graphite oxide as a support for CoSe electrocatalyst for oxygen reduction reaction in alkaline media. *J. Electrochem. Soc.* **164**, F658-F666 (2017).

34 Shi, X., Ahmad, S., Pérez-Salcedo, K., Escobar, B., Zheng, H. & Kannan, A. M. Maximization of quadruple phase boundary for alkaline membrane fuel cell using non-stoichiometric α-MnO_2 as cathode catalyst. *Int. J. Hydrogen Energy* **44**, 1166-1173 (2019).

35 Li, L., Scott, K. & Yu, E. H. A direct glucose alkaline fuel cell using MnO_2–carbon nanocomposite supported gold cata-lyst for anode glucose oxidation. *J. Power Sources* **221**, 1-5 (2013).

36 Brushett, F. R., Naughton, M. S., Ng, J. W. D., Yin, L. & Kenis, P. J. A. Analysis of Pt/C electrode performance in a flowing-electrolyte alkaline fuel cell. *Int. J. Hydrogen Energy* **37**, 2559-2570 (2012).

37 Verma, A., Jha, A. K. & Basu, S. Manganese dioxide as a cathode catalyst for a direct alcohol or sodium borohydride fuel cell with a flowing alkaline electrolyte. *J. Power Sources* **141**, 30-34 (2005).

38 Jafri, R. I., Sujatha, N., Rajalakshmi, N. & Ramaprabhu, S. Au–MnO_2/MWNT and Au–

ZnO/MWNT as oxygen reduc-tion reaction electrocatalyst for polymer electrolyte membrane fuel cell. *Int. J. Hydrogen Energy* **34**, 6371-6376 (2009).

39 Naughton, M. S., Brushett, F. R. & Kenis, P. J. A. Carbonate resilience of flowing electrolyte-based alkaline fuel cells. *J. Power Sources* **196**, 1762-1768 (2011).

40 Unni, S. M., Mora-Hernandez, J. M., Kurungot, S. & Alonso-Vante, N. $CoSe_2$ Supported on Nitrogen-Doped Carbon Nanohorns as a Methanol-Tolerant Cathode for Air-Breathing Microlaminar Flow Fuel Cells. *ChemElectroChem* **2**, 1339-1345 (2015).

41 Chen, F., Chang, M.-H. & Hsu, C.-W. Analysis of membraneless microfuel cell using decomposition of hydrogen peroxide in a Y-shaped microchannel. *Electrochim. Acta* **52**, 7270-7277 (2007).

42 Tamura, T., Ohwaki, T., Ito, A., Ohsawa, Y., Kobayashi, R. & Ogata, S. Theoretical Mn K-edge XANES for Li_2MnO_3: DFT + *U* study. *Modelling Simul. Mater. Sci. Eng.* **20**, 045006 (2012).

43 Ricca, C., Timrov, l., Cococcioni, M., Marzari, N. & Aschauer, U. Self-consistent DFT+*U*+*V* study of oxygen vacancies in $SrTiO_3$. *Phys. Rev. Research* **2**, 023313 (2020).
